# Mutation rate and spectrum in obligately outcrossing *Caenorhabditis elegans* mutation accumulation lines subjected to RNAi-induced knockdown of the mismatch repair gene *msh-2*

**DOI:** 10.1093/g3journal/jkab364

**Published:** 2021-10-21

**Authors:** Vaishali Katju, Anke Konrad, Thaddeus C Deiss, Ulfar Bergthorsson

**Affiliations:** 1 Department of Veterinary Integrative Biosciences, Texas A&M University, College Station, TX 77845, USA; 2 Faculdade de Ciência da Universidade de Lisboa (FCUL), CE3C—Centre for Ecology, Evolution and Environmental Changes, 1749-016 Lisboa, Portugal

**Keywords:** mismatch repair, *msh-2*, mutation accumulation, base substitution, small indel, *Caenorhabditis elegans*, experimental evolution, whole-genome sequencing, obligately outcrossing strain, RNAi

## Abstract

DNA mismatch repair (MMR), an evolutionarily conserved repair pathway shared by prokaryotic and eukaryotic species alike, influences molecular evolution by detecting and correcting mismatches, thereby protecting genetic fidelity, reducing the mutational load, and preventing lethality. Herein we conduct the first genome-wide evaluation of the alterations to the mutation rate and spectrum under impaired activity of the *MutSα* homolog, *msh-2*, in *Caenorhabditis elegans* male–female *fog-2(lf)* lines. We performed mutation accumulation (MA) under RNAi-induced knockdown of *msh-2* for up to 50 generations, followed by next-generation sequencing of 19 MA lines and the ancestral control. *msh-2* impairment in the male–female background substantially increased the frequency of nuclear base substitutions (∼23×) and small indels (∼328×) relative to wildtype hermaphrodites. However, we observed no increase in the mutation rates of mtDNA, and copy-number changes of single-copy genes. There was a marked increase in copy-number variation of rDNA genes under MMR impairment. In *C. elegans*, *msh-2* repairs transitions more efficiently than transversions and increases the AT mutational bias relative to wildtype. The local sequence context, including sequence complexity, G + C-content, and flanking bases influenced the mutation rate. The X chromosome exhibited lower substitution and higher indel rates than autosomes, which can either result from sex-specific mutation rates or a nonrandom distribution of mutable sites between chromosomes. Provided the observed difference in mutational pattern is mostly due to MMR impairment, our results indicate that the specificity of MMR varies between taxa, and is more efficient in detecting and repairing small indels in eukaryotes relative to prokaryotes.

## Introduction

Mutation is a ubiquitous process across organisms, introducing novel genetic variation from which adaptive processes can sample. However, when mutational events facilitated by replication errors, exogenous (UV radiation, chemicals), or endogenous (oxygen free radicals) influences, are left entirely untempered, genome integrity falls victim to this same evolutionarily essential process (Lindahl 1993; Kunkel and Erie 2005). A number of DNA repair mechanisms have evolved that limit the degenerative processes of DNA damage and replication errors. One such system is DNA mismatch repair (MMR), which corrects errors arising *via* base–base mismatches and small loop-outs resulting in indel mutations (Harfe and Jinks-Robertson 2000; Aquilina and Bignami 2001; Kunkel and Erie 2005; Jiricny 2006) by detecting the mismatch, differentiating between the parent and newly synthesized strand, and initializing repair (Modrich 1991; Kolodner 1996). MMR influences molecular evolution in various ways, most notably by detecting and correcting mismatches that escape DNA polymerase proofreading, thereby protecting genetic fidelity, reducing the mutational load, and preventing lethality. Conversely, loss-of-function mutations in MMR genes can increase the mutation rate by several orders of magnitude. Furthermore, the specificity of the MMR system, with respect to the efficiency of MMR in recognizing and repairing different kinds of mismatch errors, contribute to the evolution of the base composition and structure of genomes.

MMR systems are found in functionally conserved form across the tree of life, consistent with their essentiality and ancient origin (Kolodner 1996; Eisen and Hanawalt 1999; Kunkel and Erie 2005; Lin *et al.* 2007; Sachadyn 2010). The *Escherichia* *coli* (and other alpha-, beta-, and gamma-proteobacteria) MMR pathway contains a single pair of MutS and MutL proteins, while many other bacterial and eukaryotic genomes code for a repertoire of several MutS and MutL homolog proteins (MSH and MLH, respectively). Eukaryotic genomes encode several different MSH proteins, the sum of which perform a highly conserved MMR recognition mechanism (Groothuizen and Sixma 2016). MutS is required for the recognition of DNA lesions and recruits MutL for subsequent repair, which, in heterodimeric forms of their respective homologs, carry out the various functions of MMR (Modrich 1991; Harfe and Jinks-Robertson 2000; Kunkel and Erie 2005; Lin *et al.* 2007; Sachadyn 2010). However, the exact number of homologs and their exact functional complementation is not perfectly conserved (Denver *et al.* 2005; Groothuizen and Sixma 2016). *msh-4* and *msh-5* play important roles in chiasma formation during meiotic recombination (Shodhan *et al.* 2014), but have no known functions related to MMR (Zalevsky *et al.* 1999). The MSH2/MSH3 heterodimer (MutS*β*) corrects small and large loop-outs (no base–base MMR) in yeast, humans, and other mammals (Alani 1996; Habraken *et al.* 1996; Aquilina and Bignami 2001; Harrington and Kolodner 2007; Lee *et al.* 2007); yet *msh-3* has not been detected in several other metazoans, including *Drosophila* and *Caenorhabditis* (Tijsterman *et al.* 2002; Lin *et al.* 2007). Certain plants (*Arabidopsis thaliana*) form a MSH2/MSH7 (MutS*γ*) complex which corrects certain mismatches and loop-outs (Culligan and Hays 2000; Gómez and Spampinato 2013); however, the *msh-7* homolog has so far only been detected in plants. In contrast, the MSH2/MSH6 (MutS*α*) heterodimer complex is more strongly conserved across metazoan taxa (Lin *et al.* 2007). The MutS*α* complex is responsible for base–base mismatch and small loop-out indel detection, as well as the initiation of the repair cascade (Harfe and Jinks-Robertson 2000; Aquilina and Bignami 2001; Kunkel and Erie 2005; Richman 2015). A defect in, or insufficient concentration of the MutS*α* complexes results in microsatellite and genome instability and promotes tumorigenesis in humans (Aquilina and Bignami 2001; Degtyareva *et al.* 2002; Tijsterman *et al.* 2002; Lynch *et al.* 2009; Richman 2015; Meier *et al.* 2018).

Defects in the MutS*α* complex have been shown to lead to increased repeat tract length instability (mutation rate fold changes between 100 and 700) in multiple species, such as *Saccharomyces* *cerevisiae* and *Caenorhabditis* *elegans* (Strand *et al.* 1993, Degtyareva *et al.* 2002; Tijsterman *et al.* 2002; Denver *et al.* 2005). Additionally, base substitution rates increased between five and more than 30-fold between wildtype and knockout lines of the MutS*α* complex (Yang *et al.* 1999; Degtyareva *et al.* 2002; Tijsterman *et al.* 2002; Denver *et al.* 2005). However, the majority of these studies did not consider the genome as a whole, but rather relied on mutation rate estimates based on partial genome analysis or on specific reporter genes. For example, the Denver *et al.* (2005) analysis was restricted to 20 kb, which represents <0.02% of 100.3 Mb *C. elegans* genome. Differences in mutational rates and spectra arising due to sequence context, genome architecture, functional content, and transcription or replication cannot be captured in approaches that do not evaluate the genome as a whole. Mutation accumulation (MA henceforth) experiments coupled with whole-genome sequencing (WGS) provides an experimental framework to assess such variability (reviewed in Katju and Bergthorsson 2019). MA experiments consist of passaging independently evolving populations through single individual bottlenecks, thus rendering the effects of natural selection minute. All but the most deleterious or lethal mutations are thus allowed to accumulate as if neutral with respect to fitness, thereby facilitating a more comprehensive assessment of the mutational process. The first such experiments utilizing *msh-2* knockout mutant lines followed by next-generation sequencing in the unicellular yeast *S. cerevisiae* (Lang *et al.* 2013) and the angiosperm model plant *A. thaliana* (Belfield *et al.* 2018) demonstrated an estimated 225-fold and >1000-fold increase in the spontaneous mutation rate, respectively.

Although several preceding studies in *C. elegans* have offered insights into the mutational consequences of *msh-2* deficiency, they have been limited in their scope owing to a narrow focus on specific reporter genes and known mutational hotspots, or on a limited number of nuclear and mitochondrial loci across the genome (Degtyareva *et al.* 2002; Tijsterman *et al.* 2002; Denver *et al.* 2005). MA studies with *C. elegans msh-2* knockout mutants are challenging as they have greatly reduced fertility and *msh-2* lines that are maintained by single individual descent typically become extinct in 10–20 generations (Degtyareva *et al.* 2002). In lieu of *msh-2* knockout mutation lines, we employed a *msh-2* RNA interference (RNAi henceforth) approach to knockdown the expression of *msh-2* during the MA process. Although a knockdown may not capture the full effect of a gene knockout, it enables a more extended MA experiment which helps illuminate the kinds of mutations that accumulate with an MMR system defective in *msh-2.* Several studies have shown differences in mutation rates between males and females which can manifest as different mutation rates between the X chromosome and the autosomes. In addition to the RNAi knockdown approach, we used an obligately outcrossing (male–female) line of *C. elegans* owing to a loss-of-function mutation in the *fog-2* gene, *fog-2(q71lf).* Although these deviate from the normal reproductive mode of *C. elegans*, which is predominantly self-fertilizing hermaphroditism, the results facilitate a comparison of the number of mutations between the X chromosome and the autosomes as males are XO and females are XX. A higher mutation rate in males therefore predicts a higher mutation rate on the autosomes than on the X chromosome (Miyata *et al.* 1987). These experiments (1) provide the first genome-wide view of the MA process in *C. elegans* with an impaired *msh-2,* (2) facilitate an investigation into mutation rate differences between the sex chromosome and autosomes, and (3) present an analysis of the greatest number of mutations in any MMR-deficient background in the species.

## Materials and methods

### MA lines subjected to *msh-2* knockdown

The MA lines used in the *msh-2* knockdown experiment were established from a single male–female pair of the obligatory outcrossing *fog-2(q71lf)* mutant strain [henceforth referred to as *fog-2(q71*)] of *C. elegans* (Schedl and Kimble 1988; Katju *et al.* 2008; Farslow *et al.* 2015). Following four generations of single-pair sibling-mating, two males and one female from the F_5_ offspring of the founding pair were used to establish 74 MA lines (Supplementary Figure S1) (Katju *et al.* 2008). The remaining founder population was frozen at −80°C to be used as the ancestral control, referred to as the pre-MA ancestral control. The MA lines were subjected to repeated population bottlenecks by randomly picking one female and two male L4 larval worms to establish the subsequent generation (Supplementary Figure S1). Given a population bottleneck of three individuals (two males and one female) each generation, the effective population size, *N_e_*, is approximately 2.67 individuals (Wright 1931). Hence, the *N_e_* of this MA experiment was similar to that of our MA experiment with wildtype, facultatively outcrossing lines maintained at population bottlenecks of *N *=* *1 per generation (*N_e_* = 1; Katju *et al.* 2015, 2018). Under both these MA regimes, mutations with selection coefficients less than approximately 10% are expected to contribute to mutational degradation given that they will accumulate within these MA lines at the neutral rate, although they may not necessarily be neutral with respect to absolute fitness. However, our previous analyses of the accumulation of SNPs and small indels in *C. elegans* MA lines found no differences between MA lines due to *N_e_* (Konrad *et al.* 2019).

The expression of the *msh-2* MMR gene was knocked down each MA generation *via* a standard RNAi feeding protocol (Kamath *et al.* 2001) in order to elevate the accumulation of germline and somatic mutations (Degtyareva *et al.* 2002; Tijsterman *et al.* 2002). The gene of interest, *msh-2*, was amplified *via* PCR and cloned into the L4440 vector, which has two T7 promoters in inverted orientation flanking the cloning site. The cloned plasmids were transformed into HT115(DE3), an RNase III-deficient *E. coli* strain with IPTG-inducible expression of the T7 polymerase. A bacterial clone containing the feeding vector with the *msh-2* gene was obtained from Julie Ahringer at the University of Cambridge. Single colonies of HT115 bacteria containing cloned L440 plasmids with the *msh-2* gene were picked and cultured in LB with 50 µg/ml ampicillin for approximately 8–12 h. In order to induce the expression of dsRNA of the *msh-2* gene, these cultures were then seeded directly onto NGM plates with 1 mM IPTG and 50 µg/ml ampicillin. Seeded plates were allowed to dry at room temperature and the induction of dsRNA was continued overnight. For each generation of MA, one female and two male siblings were placed onto the freshly prepared NGM feeding plates containing seeded bacteria expressing dsRNA for the *msh-2* gene. The resulting progeny were maintained on these RNAi feeding plates for 4 days at 20°C, following which another generation of MA on new RNAi feeding plates was initiated in the manner described above. To prevent accidental losses of the experimental lines, plates from the preceding three generations were maintained as backups in a separate 20°C chamber. Each line was subjected to 50 generations of MA, with bottlenecking and RNAi treatment at each generation. MA lines that failed to produce any progeny in a particular generation of the experiment were reinitiated from backup populations as needed for a maximum number of three consecutive attempts prior to being considered formally extinct. Four lines became extinct during this MA phase due to a complete lack of recruitment of new generations of individuals, despite backup attempts from preceding generations.

It is critical that mutations accumulated in the MA phase of the experiment are fixed within each line and not capable of segregation as wildtype alleles. To achieve this, each extant MA line was subjected to 15 additional generations of full-sibling mating without RNAi treatment. Treating the last MA generation as the reference population, 15 generations of full-sibling mating should yield an inbreeding coefficient of 0.961 (*i.e.*, a 96.1% reduction in heterozygosity relative to a random-mating subpopulation with the same allele frequencies) (Falconer 1989). In practice, the reduction in heterozygosity should be greater given that the MA treatment involved a strict regime of full-sibling mating, even though a single female was paired with and could be multiply mated to two brothers. Thereafter, all surviving MA lines (total 70 lines) were frozen at −80°C. Nineteen of the extant 70 MA lines and the ancestral control were sequenced for this study. This set included five MA lines previously observed to have experienced the most extreme decline in fitness (Farslow *et al.* 2015), and 14 additional randomly chosen lines.

### Genomic DNA extraction, WGS, and alignment

A total of 20 *C. elegans fog-2(q71)* lines comprising the ancestral control and 19 *msh-2* knockdown MA lines were prepared for DNA WGS. For each line, a trio of one female and two male worms were used to establish a population that was expanded for two to three additional generations to generate sufficient worm tissue for genomic DNA (gDNA) extraction. Sequencing followed the methodologies previously described (Konrad *et al.* 2017, 2018, 2019). The PureGene Genomic DNA Tissue Kit (QIAGEN no. 158622) and a supplementary nematode protocol were used for isolation of gDNA. DNA quality and concentration were checked on 1% agarose gels through electrophoresis, a Nanodrop spectrophotometer (Thermo Fisher), and BR Qubit assay (Invitrogen). Sonication of 2 μg of each DNA sample in 85 μl TE buffer yielded target fragment lengths of 200–400 bp, which were end-repaired [NEBNext end repair module (New England BioLabs)] and purified [Agencourt AMPure XP beads (Beckman Coulter Genomics)]. Beads were removed after adapter ligation as previously described (Thompson *et al.* 2013). Custom preannealed Illumina adapters were utilized and ligated to the purified fragments. 3′ adenine overhangs were added (AmpliTaq DNA Polymerase Kit, Life Technologies). PCR amplification was performed *via* Kapa Hifi DNA Polymerase (Kapa Biosystems) and Illumina’s paired end gDNA primers containing 8 bp barcodes. Size fractionation of the PCR products was performed on 6% PAGE gels and 300–400 bp fragments were selected. Gel extraction by diffusion at 65°C and gel filtration (NanoSep, Pall Life Sciences) followed by Agencourt AMPure bead purification was used to generate the final fully purified fragments. The fragments’ final quality and quantity was checked *via* the Agilent HS Bioanalyzer and HS Qubit assays. Multiplexed libraries were sequenced on Illumina HiSeq sequencers with default quality filters at the Northwest Genomics Center (University of Washington).

The demultiplexed raw reads were aligned to the reference N2 genome (version WS247; www.wormbase.org; Harris *et al.* 2010) using the Burrows–Wheeler Aligner (BWA Version 0.5.9) (Li and Durbin 2009) and Phaster (Green laboratory) (previously described in Konrad *et al.* 2018, 2019).

### Sequence alignment and identification of putative single nucleotide polymorphisms and indels

The identification of small mutations (SNPs and indels up to 100 bp long) followed the same methodology previously used to identify mutations in the wildtype MA lines relative to their ancestral control (Konrad *et al.* 2019). Briefly, alignment files from Phaster and BWA were analyzed separately. Putative SNPs and indels were identified using Platypus (Rimmer *et al.* 2014), Freebayes (Garrison and Marth 2012), and a pipeline consisting of mpileup (Li *et al.* 2009), bcftools (Li 2011), vcfutils (Danecek *et al.* 2011), and custom filters written in Perl. All putative variants were filtered against the ancestral control line, and Indelminer (Ratan *et al.* 2015) was used as an additional approach to call indels with the ancestral control genome as a direct reference (normal sample). Putative indel identification was primarily based on the Phaster alignments due to its greater ability to split reads and align with gaps. However, BWA alignments were used to verify these indel calls. A minimum root-mean-square mapping quality of 30 and 40 was required for SNPs and indels to be retained, respectively. A minimum of three and five high-quality reads were required to support each SNP and indel, respectively. Putative variants present in the ancestral control genome, even with low-quality or coverage, were removed from the analysis. A minimum of 80% of all high-quality calls at the variant position were required to support a variant in question for it to be retained in the dataset. Finally, each variant had to be called independently by at least two of the variant callers in order to be considered for further analysis.

To rule out putative variants identified due to sequencing or alignment error, every variant was independently verified by calculating a binomial probability for it, given the number of variant calls at the same location in the genome across all other populations sequenced (Konrad *et al.* 2019). For each putative variant position, the number of reads across all lines calling the variant were summed and divided by the total number of reads at the variant position. We used this as the probability of any given read calling the variant by chance (*P*). For each putative mutation, we counted the number of reads within every individual line which called the variants (*K*), and the total number of reads at the position in that line (*N*). We then calculated the *P*value for the variant (var) in that line (*i*): ${p_{\mathrm{var}}}_{i}\;=(\frac{N!}{K!\left(N-K\right)!})\;\times \;(P^{K})\;\times \;((1-{P)}^{N\;-\;K})).\;$The *P*values across all lines were sorted from most significant to least significant, and a Holm–Bonferroni correction was applied to determine if the variants called by the previous pipeline met the critical *P-*value threshold.

### Annotation, characterization, and mutation rate calculation for SNPs and small indels

All identified variants were annotated using a custom script and the GFF file available for the N2 reference genome of *C. elegans* (version WS247; www.wormbase.org; Harris *et al.* 2010). Mutations were assigned to exons, introns, and intergenic regions, as well as to chromosomal arms, cores, and tips based on boundaries predicted by Rockman and Kruglyak (2009). The tips domains contain roughly 7% of the *C. elegans* genome and have high gene density and extremely low recombination rates. The arms domains (26%) have high recombination rate and the center domains (cores, 47%) have low recombination rates. The boundaries in the original analysis of recombinational domains were identified using segmented linear regression (Rockman and Kruglyak 2009). Mutation rates (${{µ}_{\mathrm{var}}}_{i}$) were estimated individually for each line and across genomic subdivisions as variants per base per generation (${{µ}_{\mathrm{var}}}_{i}\;=\frac{F_{\mathrm{var}}}{G*B}$), where *F*_var_ equals the number of substitutions or indels within the line, *G* refers to the number of generations, and *B*_total_ refers to the number of bases in the genome or genomic subdivision that meet the same thresholds as for variant identification (version WS247). For mitochondrial mutation rates, the frequencies of variants were calculated as a percentage of quality reads calling the variant. Overall mutation rates were calculated by averaging the line-specific mutation rates within: $\mu_{N}\;=\frac{\sum_{i=1}^{n}\;{{µ}_{\mathrm{var}}}_{i}}{n}$, where var_*i*_ refers to the line-specific mutation rate, and *n* refers to the total number of lines. The number of MA generations through which each population was propagated differed between the lines (Supplementary Table S1).

Genomic repeat regions and homopolymeric runs were identified using the Imperfect Microsatellite Extractor (IMEX 2.1; Mudunuri and Nagarajaram 2007). Homopolymeric runs of at least 6 bp in length were included. For di- and tri-nucleotide repeats, we required at least four repetitions of the motif, while three repeats of each individual repeat unit were required for tetra-, penta-, and hexa-nucleotide repeats. Imperfect repeats were not included in the analysis of repeats, unless the imperfect repeat divided the overall repeat region into at least one run that met the above criteria. Every putative variant was mapped against this final list of genome-wide repeats.

Every protein-coding gene was categorized as either a germline or nongermline expressed gene based on the data of Wang *et al.* (2009). The mutation rate in germline expressed genes was calculated by summing the number of mutations within each line that mapped to germline expressed genes and dividing by the total number of high-quality bases within germline expressed genes. Mutation rates for nongermline expressed genes were calculated in the same fashion.

Sequence complexity was calculated as described in Morgulis *et al.* (2006). Briefly, given a sequence (*a*) of length *n* and 64 possible triplets of {A, C, G, T}, the occurrence of each possible triplet (*t*) was counted across the sequence and yields *c_t_(a)*. The total number of overlapping triplets occurring in any sequence (*l*) equals *n*-2. Sequence complexity (*S(a)*) was then calculated as:

$$S\left(a\right)=\;\frac{\sum_{t\;\in R}c_{t}(a)(c_{t}\left(a\right)-1)/2}{\left(l-1\right)}.$$

All statistical tests were performed in R (R Core Development Team 2014).

### Genomic properties influencing mutation rates

A regularized logistic regression approach was used to determine the genomic properties most indicative of the mutability of different sites in the genome (Ness *et al.* 2015; Konrad *et al.* 2019). The training set for the modeling consisted of 1,000,000 random nonmutated sites throughout the *C. elegans* genome combined with the 3,125 unique substitution sites. Chromosomal location, functional properties, germline expression (Wang *et al.* 2009), recombination rate (Rockman and Kruglyak 2009), G + C-content and sequence complexity (*s*; Morgulis *et al.* 2006), repeat sequence, chromatin state (Evans *et al.* 2016), periodic A_*n*_/T_*n*_-clusters (Frøkjær-Jensen *et al.* 2016), and trinucleotide sequence context for each of the 1,003,125 sites (∼1% of the genome, each) were compiled as potential predictors for mutability (Konrad *et al.* 2019). G + C-content and sequence complexity (*s*) were calculated for 41 bp windows around each site (Konrad *et al.* 2019). All categorical predictors (chromosome, functional category, trinucleotide context) were converted to a series of binary predictors referring to each category level. Recombination rate, G + C-content, and sequence complexity were treated as numeric predictors, while germline expression and repeat sequence were binary predictors.

The GLMnet package (v1.9-8) was used to perform a generalized linear model fit in R (R Core Development Team 2014). This package implements penalized maximum likelihood through ridge and lasso regression, resulting in more precise fits for models which are built with intercorrelated predictor variables (Friedman *et al.* 2010). The response variable was binary: 1 for a mutation and 0 for a random site/no mutation. The penalty against significant correlations between predictor coefficients was determined by the regularization parameter (λ). λ was set to 6.83 × 10^−5^, which is the value (lambda.min) at which the crossvalidated error is minimized through the built-in crossvalidation function during the model building step. An *α* of 0.01 was used to retrieve the model coefficients, which shrinks correlated predictor coefficients together. Varying *α* did not affect the model fit. Odds ratios (OR) for predictors were calculated as OR = *e^c^*, where *c* refers to a given predictor coefficient.

For each site in the genome, its mutability was estimated as the probability of encountering a mutation, using the predict function of GLMnet with the model coefficients estimated above for each genomic predictor. The probabilities of mutation at any given site are affected by the proportion of mutated sites over random sites used during the model training step. Hence, we are interested in the relative mutability values. Given the 3,125 SNP sites (or 9,858 unique indel sites) and 1,000,000 nonmutated sites, the mean predicted mutability is approximately 0.002. The predicted mutability across the genome ranged from 0.75 × 10^−4^ to 0.57. One hundred percent of SNPs were covered between mutabilities of 0.0 and 0.68 for SNPs. Mutabilities from 0 to 0.12 (encompassing > 99.9% of SNPs and >99.9% of genomic sites) were combined into bins of size 0.015, and mutation rates were calculated for each bin: $=\frac{\mathrm{SNPb}}{B\;x\;G}$, where *SNP_b_* refers to the number of SNPs per bin, *B* refers to the number of sites in each bin, and *G* refers to the average number of generations (40.3). Correlation coefficients and *R*^2^ values were calculated for a linear (Pearson) regression of mutation rate over mutability in R (R Core Development Team 2014).

## Results

We sequenced the genomes of the *fog-2(q71)* ancestral control and 19 descendant MA lines subjected to *msh-2* knockdown *via* RNAi with an average read depth of 30.35× and 16.51×, respectively (Supplementary Figure S1 and Table S1). Although each experimental line was subjected to 50 consecutive rounds of population bottlenecks and *msh-2* RNAi, the MA generation numbers for this set of experimental lines ranged from 28 to 45 due to the frequent need for reinitiation of experimental lines from backup generations, with an average of 40.3 MA generations for this set of 19 MA lines. The proportion of the genome included for SNP analysis across the experimental *fog-2(q71)*; *msh-2*(RNAi) knockdown MA lines ranged from 95 to 97%. More stringent filtering thresholds imposed for the indel analysis led to the inclusion of 59–69% of the genome. The whole-genome sequences of the ancestral control and MA lines can be accessed through the National Center for Biotechnology Information Sequence Read Archive (Bioproject PRJNA554105).

Prior to combining the mutation rate data for the five lowest fitness MA lines with the additional randomly chosen 14 MA lines comprising our dataset, we tested if there was a difference in the base substitution and the indel rates between these two groups. The average base substitution rate for the five low fitness and 14 randomly chosen *fog-2(q71)*; *msh-2*(RNAi) MA lines was 3.99 × 10^−8^/site/generation and 4.30 × 10^−8^/site/generation, respectively. The slightly higher value for the randomly chosen MA lines was not significantly different from the MA lines with the lowest fitness (*t *=* *0.89, *P* *= *0.38). The results for small indels were very similar. The small indel rate for the low fitness and the randomly chosen MA lines was 2.02 × 10^−7^/site/generation and 2.31 × 10^−7^/site/generation, respectively. The difference in indel rates was not significant (*t *=* *1.54, *P* *= *0.14). Given that the two mutation rates are essentially equivalent, we used the combined dataset of our 19 *fog-2(q71)*; *msh-2*(RNAi) MA lines for all downstream analyses.

### Significantly elevated nuclear mutation rates in obligately outcrossing *msh-2* knockdown MA lines relative to wildtype (selfing) MA lines

**Figure 1:**
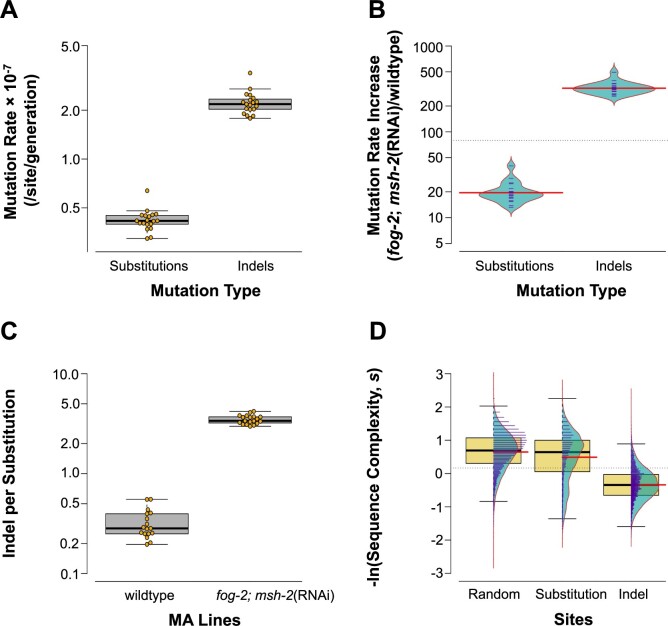
Increase in mutation rates in *fog-2(q71)*; *msh-2*(RNAi) knockdown MA lines compared with wild type. (A) Base substitution rates are significantly lower than small indel rates in the obligately outcrossing *msh-2* knockdown lines. Rates are calculated as per site per generation. (B) Mutation rate increase in obligately outcrossing *msh-2* knockdown MA lines compared with wildtype MA lines is significantly greater for small indels compared with SNPs*.* For brevity, *fog-2*; *msh-2*(RNAi) in the figures refers to *fog-2(q71)*; *msh-2*(RNAi) MA lines. (C) The indels/substitution ratio is significantly higher in the obligately outcrossing *msh-2* knockdown MA lines compared with wildtype MA lines. (D) Sequence complexity in the vicinity of indel mutations is significantly lower than that for SNPs or randomly chosen genomic sites. Sequence context in the vicinity of SNPs is significantly lower than that for random sites. Thick horizontal black lines and red lines indicate the sample median and mean, respectively.

The vast majority of mutations identified in the *fog-2(q71)*; *msh-2*(RNAi) MA lines were small indels and single nucleotide substitutions in the nuclear genome (Supplementary File S1). We identified 3,125 substitutions and 10,861 small indels across these 19 *fog-2(q71)*; *msh-2*(RNAi) knockdown MA lines following an average of only 40.3 MA generations, resulting in a combined mutation rate of 2.65 × 10^−7^ mutations per site per generation. This yields a nuclear substitution and indel rate of 4.22 (95% CI: ± 0.30) × 10^−8^ and 2.23 (95% CI: ± 0.17) × 10^−7^ mutations per site per generation, respectively (Table  1; Figure  1A). We compared these mutation rates with our preceding analyses of hermaphroditic (selfing) spontaneous MA lines of *C. elegans* maintained at population bottlenecks of *N *=* *1 individual per generation (Konrad *et al.* 2019). We henceforth refer to the hermaphroditic spontaneous MA lines as wildtype as they represent the spontaneous mutational input in the absence of selection but under a functional DNA-repair regime and the standard mode of reproduction for this species. Relative to wildtype, the mutation rates of the *msh-2* MA lines exhibit a ∼23-fold and a ∼328-fold increase in the base substitution and small indel rate, respectively (Table  1; Figure  1B). Relative increase in mutation rate in *fog-2(q71)*; *msh-2*(RNAi) knockdown MA lines compared with wildtype MA lines is significantly greater for small indels compared with SNPs (*t*-test: *t = *24.43, *P *=* *1.61 × 10^−15^). The significantly higher frequency of indels relative to substitutions in the *fog-2(q71)*; *msh-2*(RNAi) knockdown MA lines (*t*-test: *t =* −20.92, *P = *1.18 × 10^−14^) translates into a ratio of 3.85 indels per substitution (Figure  1C), which is a significantly higher ratio than that observed in the wildtype lines (0.32 indels per SNP; Figure  1C;*t*-test: *t = *12.92, *P = *6.92 × 10^−10^).

**Table 1:** ** Mutation rates (*µ* × 10^−7^) and fold-changes in *fog-2(q71)*; *msh-2*(RNAi) MA lines (this study) in comparison to selfing MA lines of the laboratory *N2* Bristol strain (wildtype) of *C. elegans* (Konrad *et al.*** 2017, 2018, 2019**)**

Mutational class	*wt* (*µ* × 10^−7^)	*fog-2; msh-2*(RNAi) (*µ* × 10^−7^)	*µ_fog-2; msh-2_*_(RNAi)_/*µ_wt_*	Significance
*µ*_SNP_	0.0184	0.42	22.93	*
*µ*_Indel_	0.0068	2.23	327.94	*
*µ*_Del_	0.0051	1.47	288.24	*
*µ*_Ins_	0.0018	0.76	422.22	*
*µ*_mt_	1.05	2.18	2.08	–
*µ*_CNV+_	26.40	10.70	0.41	–
*µ*_CNV-_	11.90	3.95	0.33	–

Substitution (SNP), small insertion and deletion (Indel), small deletion (Del) and insertion (Ins), and mitochondrial (mt) rates are given as mutations per site per generation. Copy-number gains (CNV+) and losses (CNV−) are listed as changes per protein-coding gene per generation. Line 1T of the wildtype lines was not included as it included unusually large copy-number changes indicative of extensive chromosomal rearrangements (Konrad *et al.* 2018). Statistically significant differences in rates between the two data sets are indicated by asterisks. For brevity, *wt* and *fog-2*; *msh-2*(RNAi) refer to wildtype and *fog-2(q71)*; *msh-2*(RNAi) MA lines, respectively.

Low complexity DNA sequence repeats have a significant effect on the mutation pattern (Morgulis *et al.* 2006; Figure  1D; ANOVA: *F = *9117, *P *<* *2 × 10^−16^). The complexity in the vicinity of the small indels was significantly lower than that associated with either base substitutions (Figure  1D; Tukey’s Multiple Comparison: *P* *= *0.00; *t*-test: *t = *59.12, *P *<* *2.2 × 10^−16^) or with random sites in the genome (Tukey’s Multiple Comparison: *P* *= *0.00; *t*-test: *t = *136.99, *P *<* *2.2 × 10^−16^). Moreover, the sequence context around base substitutions was less complex than that observed in the vicinity of random sites (Tukey’s Multiple Comparison: *P* *= *0.00; *t*-test: *t = *10.75, *P *<* *2.2 × 10^−16^).

### Base substitutions accumulate neutrally within exons

The synonymous [3.97 (95% CI: ± 0.70) × 10^−8/^site/generation] and nonsynonymous [4.46 (95% CI: ± 0.36) × 10^−8^/site/generation] substitution rates were not significantly different from one another in our obligately outcrossing *msh-2* knockdown MA lines (Supplementary Figure S2A; *t*-test: *t *=* *1.21, *P* *= *0.24). Furthermore, the relative increase in synonymous and nonsynonymous substitutions in these MA lines relative to wildtype MA lines were not significantly different (*t*-test: *t *=* *1.59, *P* *= *0.13). Although the average nonsynonymous/synonymous substitution ratio (*K_a_/K_s_*) for the obligately outcrossing *msh-2* knockdown MA lines appeared to be lower than that of wildtype MA lines, there was no significant difference between them (*t*-test: *t *=* *1.23, *P* *= *0.23). Furthermore, the average *K_a_/K_s_* in our obligately outcrossing *msh-2* knockdown MA lines was not significantly different from unity, which is consistent with negligible purifying selection in exons during the MA experiment (*t*-test: *t *=* *1.83, *P* *= *0.09). Frameshift mutations [1.04 (95% CI: ± 0.09) × 10^−8^/site/generation] were less frequent than either synonymous (*t*-test: *t *=* *6.00, *P *=* *1.00 × 10^−5^) or nonsynonymous substitutions (*t*-test: *t *=* *18.03, *P *=* *5.09 × 10^−14^) (Supplementary Figure S2A). However, the increase in the rate of frameshift mutations (50×) in the obligately outcrossing *msh-2* knockdown MA lines compared with wildtype was significantly greater than the increase in either the synonymous (29×; *t*-test: *t *=* *5.98, *P *=* *7.75 × 10^−7^) or nonsynonymous (24×; *t*-test: *t *=* *10.08, *P *=* *3.08 × 10^−10^) substitution rate (Supplementary Figure S2B).

### Substitution bias in obligately outcrossing *msh-2* knockdown MA lines

**Figure 2:**
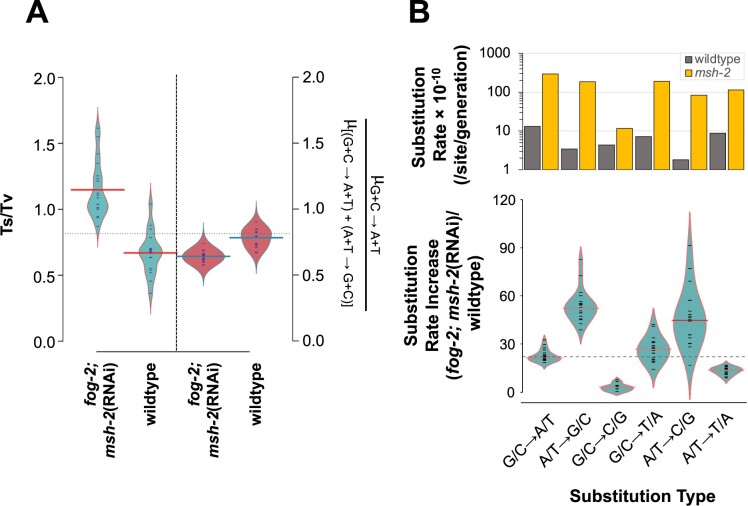
Changes in the mutational spectrum in *fog-2(q71)*; *msh-2*(RNAi) knockdown *vs* wildtype MA lines. (A) Transition to transversion (Ts/Tv) ratios are significantly greater for obligately outcrossing *msh-2* knockdown than for wildtype lines. Obligately outcrossing *msh-2* knockdown lines exhibit a significantly lower G + C → A + T mutational bias relative to wildtype MA lines. (B) The frequency of A + T → G + C substitutions shows a greater increase than G + C → A + T changes in the obligately outcrossing *msh-2* knockdown MA lines relative to wildtype. For brevity, *fog-2*; *msh-2*(RNAi) in the figures refers to *fog-2(q71)*; *msh-2*(RNAi) MA lines.

Transitions outnumbered transversions in our *fog-2(q71)*; *msh-2*(RNAi) knockdown MA lines, while the opposite was observed in the wildtype MA lines (Konrad *et al.* 2019). Consequently, the transition to transversion ratio (Ts/Tv, henceforth) was significantly higher for the obligately outcrossing *msh-2* knockdown (mean = 1.15) than for wildtype (mean = 0.67) MA lines (Figure  2A;*t*-test: *t = *7.69; *P = *6.84 × 10^−9^). There was a lower mutational bias toward A + T during *msh-2* knockdown in the obligately outcrossing MA lines than in the wildtype MA lines (Figure  2A;*t*-test: *t = *7.36, *P = *8.75 × 10^−8^). In the *fog-2(q71)*; *msh-2*(RNAi) knockdown MA lines, 64% of the base substitutions that change the G + C-content are toward an increase in A + T-content, whereas in wildtype N2 MA lines, this proportion was 78%. The increase in substitution rates in the *fog-2(q71)*; *msh-2*(RNAi) knockdown MA lines compared with wildtype varied significantly between substitution types (Figure  2B; ANOVA: *F *=* *36.41, *P *<* *2 × 10^−16^). The greatest increase in specific substitution rates between *fog-2(q71)*; *msh-2*(RNAi) knockdown and wildtype MA lines were in A/T → G/C transitions (54×) and A/T → C/G transversions (46×). In contrast, G/C → C/G transversions increased the least (3×).

### Sequence context influences obligately outcrossing mutation rates in the *msh-2* knockdown MA lines

A/T → T/A transversions exhibited the greatest context-dependence (Supplementary Figure S3). While A/T → T/A transversions in both wildtype and *fog-2(q71)*; *msh-2*(RNAi) knockdown MA lines occurred disproportionately between neighboring A and T nucleotides, the exact context-dependence differed between the two experiments. In the *fog-2(q71)*; *msh-2*(RNAi) knockdown MA lines, the strongest context-dependence was for mutations between 5′-AAT-3′ and 5′-ATT-3′ (Supplementary Figure S3A; base of focal mutation is underlined), while it was strongest for mutations between 5′-TAA-3′ and 5′-TTA-3′ in the N2 wildtype lines (Konrad *et al.* 2019; Supplementary Figure S3B). There is a pronounced difference in the effects of A/T → T/A transversions in the *fog-2(q71)*; *msh-2*(RNAi) knockdown MA lines, between 5′-A and 3′-T on one hand (19-fold increase in exons, 152-fold increase in noncoding DNA) and 5′-T and 3′-A on the other (no increase in exons, threefold increase in noncoding DNA, Supplementary Figure S3C). The frequency of C/G → T/A transitions were especially increased in exons when the focal C was positioned between two cytosines (Supplementary Figure S3C). The same was true for A/T → C/G transversions when the focal A was positioned between a 5′-A and 3′-G context in both exons and noncoding DNA (Supplementary Figure S3C). In intergenic regions, this same substitution experienced a higher than usual increase in mutation rate when the focal A was flanked by a 5′-A and a 3′-non-A nucleotide (Supplementary Figure S3C).

**Figure 3:**
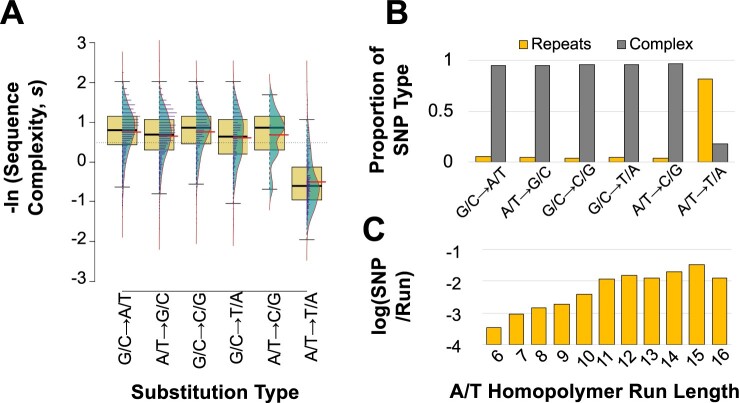
Relationship between sequence complexity and length of homopolymeric runs with individual substitution rates of *fog-2(q71)*; *msh-2*(RNAi) knockdown MA lines. (A) Significantly lower DNA sequence complexity of regions flanking A/T → T/A substitutions relative to all other substitution types. Thicker horizontal black and red lines show the sample median and mean, respectively. (B) A/T → T/A substitutions are significantly more frequent in homopolymeric runs (length ≥ 6 bp) than other substitution types. (C) The number of A/T → T/A mutations per A or T repeat (normalized by number of respective repeats across the genome) are positively correlated with homopolymer length.

A/T → T/A transversions were particularly common at the boundaries of homopolymeric runs of As and Ts. These types of homopolymeric runs are common in introns and intergenic regions, but not in exons. Furthermore, sequence complexity was significantly lower in the vicinity of A/T → T/A transversions relative to all other substitution types (Table  2; Figure  3A; ANOVA: *F *=* *427.7, *P *<* *2 × 10^−16^; Tukey’s Multiple Comparisons: *P* *= *0.00 for all pairwise comparisons between A/T → T/A and other substitution types). 80% of all substitutions occurred in complex sequence. In contrast, only 18% of A/T → T/A transversions fell within complex sequence, while 82% of these mutations were adjacent to repetitive sequence (Table  2; Figure  3B). In fact, 72% of all substitutions falling within repeat sequences were A/T → T/A transversions (Figure  2B). These repetitive sequences are almost exclusively homopolymeric A or T runs that frequently flank A/T → T/A transversions. Finally, the frequency of SNPs adjacent to A and T homopolymeric runs was positively correlated with the length of the homopolymeric run (Figure  3C; Pearson Correlation Coefficient *r *=* *0.93, *P *=* *3.33 × 10^−5^).

**Table 2:** The majority of substitutions fall within complex sequence, with the exception of A/T → T/A substitutions, which are primarily within A + T-rich repeat regions of the genome

Substitution type	Total	Complex sequence	Repeat sequence	% Repeat	% Complex
A/T → G/C	886	832	54	0.06	0.94
A/T → T/A	545	98	447	0.82	0.18
A/T → C/G	403	367	36	0.09	0.91
G/C → A/T	768	708	60	0.08	0.92
G/C → T/A	494	472	22	0.04	0.96
G/C → C/G	29	27	2	0.07	0.93
Total	3125	2504	621	0.20	0.80

### Genomic heterogeneity in the substitution rate

**Figure 4:**
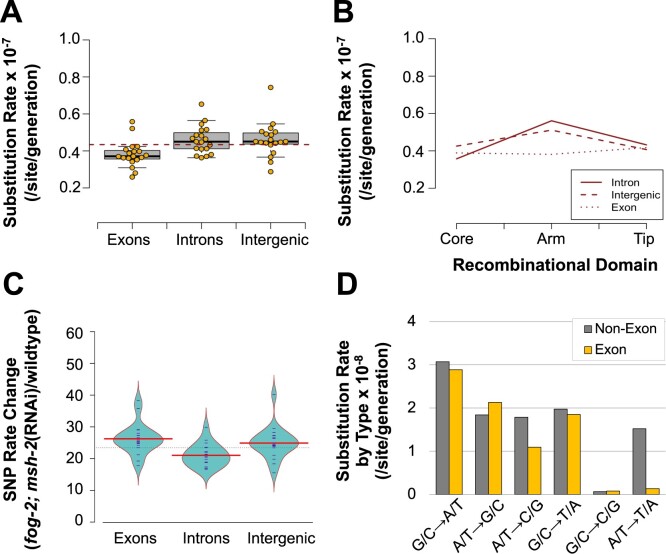
Association between coding content with the rate and spectrum of substitutions in *fog-2(q71)*; *msh-2*(RNAi) knockdown MA lines*.* (A) Exons appear to be associated with lower substitution rates than noncoding DNA. However, when location in recombinational domains (cores, arms, and tips) and chromosomes are taken into account, coding content does not have a significant influence on the substitution rates. (B) There is a significant interaction between the coding content (exon, intron, and intergenic region) and the recombinational domains. (C) The increase in substitution rates in obligately outcrossing *msh-2* knockdown MA lines relative to wildtype MA lines is significantly associated with coding content. The substitution rate increase in introns is significantly less than that in exons and intergenic regions while no difference in substitution fold-changes was observed between exons and intergenic regions. For brevity, *fog-2*; *msh-2*(RNAi)in the figures refers to *fog-2(q71)*; *msh-2*(RNAi) MA lines. (D) The mutational spectrum of exons is compared with that of nonexonic DNA (introns and intergenic regions). A ↔ T mutations are markedly lower in exons than the rest of the genome whereas C ↔ G mutations occur at extremely low rates throughout the genome.

The base substitution rate in exons, introns, and intergenic regions was 3.82 (95% CI: ± 0. 32), 4.60 (95% CI: ± 0.34), and 4.60 (95% CI: ± 0.42) × 10^−8^ substitutions/site/generation, respectively (Figure  4A). Despite the apparent low substitution rates in exons relative to noncoding DNA, the differences were not significant when their location in the genome was taken into account (3-way ANOVA: *F = *2.19, *P* *= *0.11). There is a significant interaction between the substitution rates in exons, introns, and intergenic regions with their location in cores and arms (Figure  4B; 3-way ANOVA: *F *=* *2.75, *P* *= *0.03). While there is no apparent difference based on coding content in chromosomal cores, the substitution rate in exons is 71% of that observed in noncoding DNA (introns and intergenic regions) in the chromosomal arms (Figure  4B). Coding content significantly influences the difference in substitution rates between *fog-2(q71)*; *msh-2*(RNAi) knockdown and wildtype MA lines (Figure  4C; ANOVA: *F *=* *6.86, *P* *= *0.002). Exons and intergenic regions exhibit significantly greater increase in substitution rates relative to introns (exons *vs* introns: Tukey’s Multiple Comparisons, *P* *= *0.002; intergenic *vs* introns: Tukey’s Multiple Comparisons, *P* *= *0.027). However, there was no difference in the substitution rate increase observed in exons *vs* intergenic regions (Tukey’s Multiple Comparisons: *P* *= *0.63). Higher substitution rates in noncoding DNA relative to exons is best attributed to the much higher frequency of context-dependent A/T to T/A transversions in the former (Figure  4D). These substitutions occur predominantly at the ends of homopolymeric runs of As and Ts, which are less common in exons. When A/T to T/A transversions are excluded from the analysis, there is no difference in the substitution rates between exons, introns, and intergenic regions (ANOVA: *F *=* *0.08, *P* *= *0.92).

**Figure 5:**
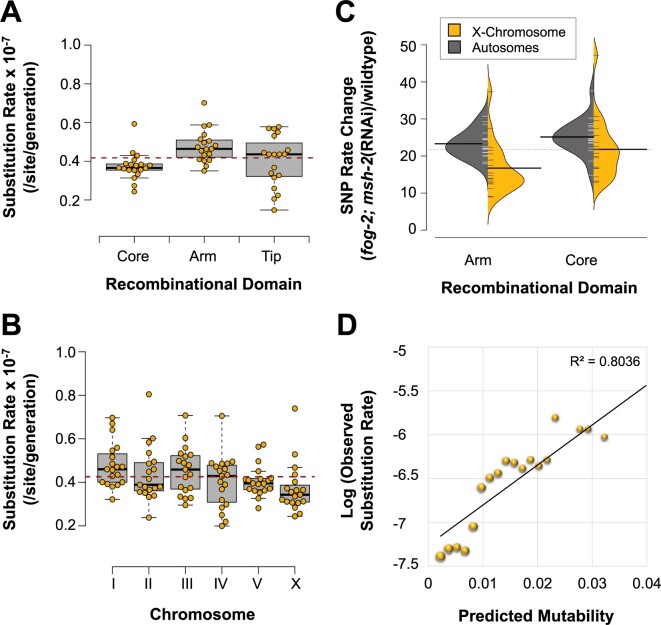
Association between genomic location and substitution rate in *fog-2(q71)*; *msh-2*(RNAi) knockdown MA lines. (A) Significant correlation between recombinational domain (arms, cores, and tips) and substitution rates. (B) Significant variation in substitution rates among chromosomes. Furthermore, autosomes have higher substitution rates than the X chromosome. (C) The increase in substitution rates in the obligately outcrossing *msh-2* knockdown MA lines relative to wildtype MA lines is greater for the autosomes than the X chromosome, and was detected in both the chromosomal arms and cores. The distribution of the increase in substitution rates for the autosomes and X chromosome is shown in gray and yellow, respectively. For brevity, *fog-2*; *msh-2*(RNAi)in the figures refers to *fog-2(q71)*; *msh-2*(RNAi) MA lines. (D) Three variables related to sequence context (repeat sequence, sequence complexity, and local G + C-content flanking a site) explain 80% of site mutability.

The base substitution rate in chromosomal cores, arms, and tips is 3.74 (95% CI: ± 0.32), 4.77 (95% CI: ± 0.38) and 4.01 (95% CI: ± 0.59) × 10^−8^ substitutions/site/generation, respectively (Figure  5A). The variation between recombinational domains comprising chromosomal cores, arms, and tips is significant (3-way ANOVA: *F *=* *5.40, *P* *= *0.0047). However, the relative increase in base substitution rates in the *fog-2(q71)*; *msh-2*(RNAi) knockdown MA lines compared with wildtype does not vary between arms, cores and tips (ANOVA: *F = *1.67, *P* *= *0.19). In contrast to our previous results for wildtype MA lines, the substitution rates in *fog-2(q71)*; *msh-2*(RNAi) knockdown MA lines were significantly different between chromosomes (Figure  5B; 3-way ANOVA: *F = *2.70, *P* *= *0.02). In particular, the X chromosome has a lower substitution rate of 3.71 (95% CI: ± 0.51) × 10^−8^ relative to the autosomal rate of 4.33 (95% CI: ± 0.28) × 10^−8^ substitutions/site/generation (paired *t*-test: *t = *3.28, *P* *= *0.004). Lower substitution rates on the X chromosome relative to the autosomes were detected both in chromosomal cores and arms (Figure  5C) (2-way ANOVA: *F *=* *12.53, *P *=* *7.05 × 10^−4^).

### Predictors of mutability

Most genomic features (chromosomal location, coding state, recombination domain, rate, and germline expression) were poor predictors of mutability in the *fog-2(q71)*; *msh-2*(RNAi) knockdown MA lines using a regularized logistic regression approach (Supplementary Figure S4A and Table S2). The presence of sequence repeats (OR = 4.42) was the best predictor for mutability (Supplementary Figure S4A; Konrad *et al.* 2019), as were certain nucleotide triplets surrounding the focal site. Local G + C-content negatively affected the mutability in the *fog-2(q71)*; *msh-2*(RNAi) knockdown MA lines (OR = 0.88). However, this effect was much less than that observed in the wildtype MA lines (Supplementary Figure S4A). Nonetheless, the strongest positive effects of nucleotide triplets on the substitution rate in the *fog-2(q71)*; *msh-2*(RNAi) knockdown MA lines were detected when C and G focal bases were flanked by C and G nucleotides (5′-GCC-3′/5′-GGC-3′ and 5′-CGC-3′/5′-GCG-3′; OR = 4.90 and 4.11, respectively). The relationship between several nucleotide triplets and mutability differed between the *fog-2(q71)*; *msh-2*(RNAi) knockdown and wildtype MA lines, with some shifting from positive effects to negative effects and *vice versa* (Supplementary Figure S4A). With the exception of 5′-AAT-3′/5′-ATT-3′ (OR = 2.22), all other A + T triplets had negligible or negative effects on the mutability of a site (Supplementary Table S2 and Figure S4A). Most triplets containing at least two-thirds C + G bases increased mutability, while most triplets with two-thirds A + T bases reduced mutability.

An average mutability of 0.04 encompassed 100% of all SNPs and 99.9% of all genomic sites (Supplementary Figure S4B). The model can account for 78.8% of the variance in mutability using the same predictors as previously employed for wildtype MA lines (Supplementary Figure S4C; Konrad *et al.* 2019). However, 80.4% of the mutational variance could be accounted for if only a subset of three predictors related to sequence context (sequence repeat, G + C-content, and sequence complexity) were used to predict mutability (Figure  5D).

### Variation in indel rates across the genome

**Figure 6:**
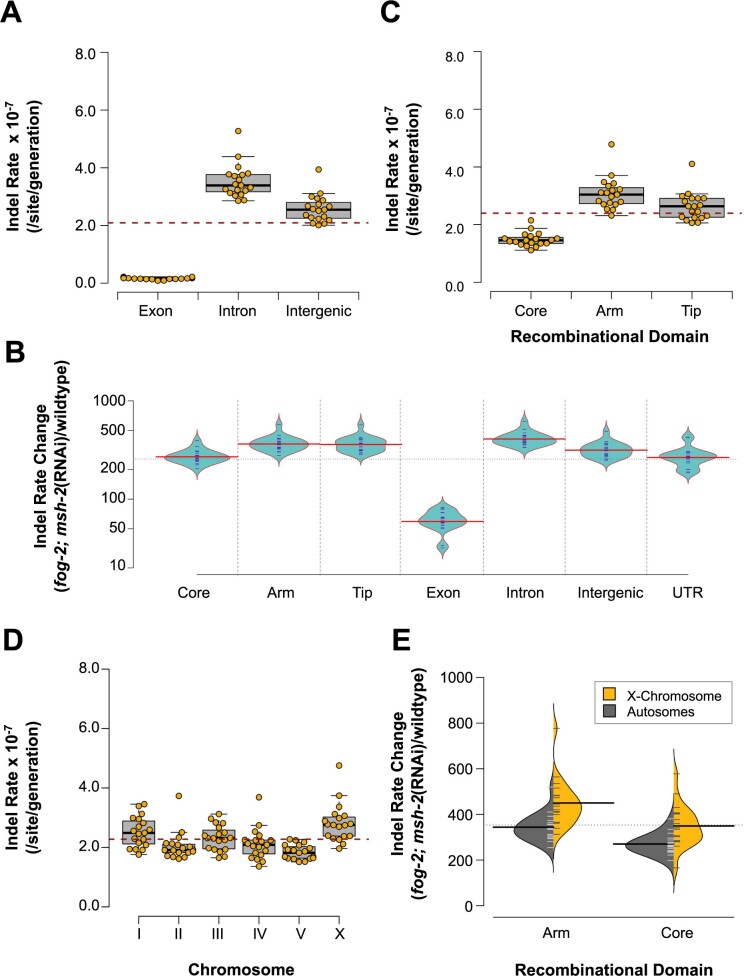
Association between genomic location and small indel rate in *fog-2(q71)*; *msh-2*(RNAi) knockdown MA lines. For brevity, *fog-2*; *msh-2*(RNAi) in the figures refers to *fog-2(q71)*; *msh-2*(RNAi) MA lines. (A) Indel rates differ significantly with coding content. (B) The relative increase in indel rates in obligately outcrossing *msh-2* knockdown MA lines relative to wildtype MA lines varies significantly between recombinational domains and coding content. While the relative increase in indel rate is significantly greater in arms and tips than in cores, the relative increase is not significantly different between arms and tips. The relative increase in the indel rate in the obligately outcrossing *msh-2* knockdown MA lines is significantly less in exons than noncoding DNA. (C) Indel rates differ significantly with recombinational domain. (D) Indel rates varied significantly by chromosome, and between autosomes and the X chromosome. (E) The relative increase in the indel rate in the obligately outcrossing *msh-2* knockdown MA lines relative to the wildtype MA lines was significantly greater in the X chromosome than in the autosomes and was observed on both arms and cores.

The indel rates observed in the *fog-2(q71)*; *msh-2*(RNAi) knockdown MA lines were dependent on genomic location (Figure  6, A–C). Exons had lower indel rates than introns and intergenic regions (Figure  6A; three-way ANOVA: *F *=* *886.82, *P *<* *2.0 × 10^−16^). Additionally, rate changes during *msh-2* knockdown relative to the wildtype MA lines differed significantly between exons and noncoding DNA (Figure  6B; ANOVA: *F = *135.8, *P *<* *2 × 10^−16^). The increase in indel rates was substantially greater in introns and intergenic regions relative to exons (Tukey’s Multiple Comparisons: introns *vs* exons, *P = *0.00; intergenic regions *vs* exons regions, *P* *= *0.00). These differences between exons and noncoding DNA are likely due to differential sequence complexity and composition in exons relative to introns and intergenic regions, as exons have higher G + C-content and fewer homopolymeric A/T runs.

Similarly, chromosomal cores had lower indel rates than arms and tips (Figure  6C; 3-way ANOVA: *F *=* *108.67, *P *<* *2.0 × 10^−16^). Furthermore, the relative increase in indel rates during *msh-2* knockdown relative to the wildtype MA lines was greater in the arms and tips relative to the cores (Figure  6B; ANOVA: *F = *15.75, *P *<* *4.09 × 10^−6^; Tukey’s Multiple Comparisons: *P* *= *0.00). The distribution of indels across the recombinational regions of the chromosome was not significantly different between deletions and insertions (Fisher’s Exact Test: *P* *= *0.47). As discussed above for exons, genedense cores harbor a lower proportion of mutation-prone A/T homopolymeric runs than do arms, and hence the indel rates in cores did not increase as much as in arms.

The distribution of indels showed significant variation across chromosomes (Figure  6D; three-way ANOVA: *F = *4.22, *P = *8.41 × 10^−4^). In contrast to nucleotide substitutions, the X chromosome appears to have a higher indel rate than the autosomes (*t*-test: *t = *4.06, *P = *3.68 × 10^−4^). Furthermore, the increase in the indel rate on the X chromosome was greater than that on the autosomes in the *fog-2(q71)*; *msh-2*(RNAi) knockdown relative to the wildtype MA lines, and this difference was consistent across both cores and arms (Figure  6E; two-way ANOVA: *F *=* *26.55, *P *=* *2.16 × 10^−6^).

### Deletions outnumber insertions and a preponderance of single base indels

*fog-2(q71)*; *msh-2*(RNAi) knockdown MA lines have significantly elevated indel rates relative to wildtype MA lines (Figure  1B;*t*-test: *t = *26.10, *P = *9.28 × 10^−16^), but the pattern of relative increase differs between insertions and deletions. The average deletion rate of 1.47 (95% CI: ± 0.13) × 10^−7^/site/generation is significantly higher than the average insertion rate of 7.59 (95% CI: ± 0.50) × 10^−8^/site/generation in the *fog-2(q71)*; *msh-2*(RNAi) knockdown MA lines (Supplementary Figure S5A; *t*-test: *t = *10.07, *P = *5.75 × 10^−10^). While the wildtype MA lines contained an average of 3.75 deletions for each insertion, *fog-2(q71)*; *msh-2*(RNAi) knockdown MA lines had a significantly lower ratio of 1.94 (Supplementary Figure S5B; *t*-test: *t = *2.61, *P* *= *0.019). The average increase in the insertion rate during *msh-2* knockdown compared with wildtype lines is significantly higher (422×) than that for deletions (288×) (Supplementary Figure S5C; *t*-test: *t = *6.89, *P *=* *4.84 × 10^−8^). In the *fog-2(q71)*; *msh-2*(RNAi) knockdown MA lines, the shortest indels (1–2 bp in length) exhibited the greatest increase in mutation rate relative to wildtype MA lines (three-way ANOVA: *P *<* *2.0 × 10^−16^ for type, size, and the interaction of the two) and indel rate fold changes between *fog-2(q71)*; *msh-2*(RNAi) knockdown MA lines and the wildtype (Supplementary Figure S5D; three-way ANOVA: *P *<* *2.0 × 10^−16^ for size, *P *=* *2.84 × 10^−12^ type, and *P *=* *1.03 × 10^−10^ for the interaction between size and type). While the frequency of ≥3 bp indels are increased 26×, 1–2 bp indels are increased 446× (Supplementary Figure S5D).

Supplementary Figure S6 compares the size distributions of small indels in the *fog-2(q71)*; *msh-2*(RNAi) knockdown and wildtype MA lines (Konrad *et al.* 2019). There was no difference in the average size of insertions (1.06 bp) and deletions (1.08 bp) in the *fog-2(q71)*; *msh-2*(RNAi) knockdown MA lines (*t*-test: *t = *1.32, *P* *= *0.19). However, the overall indel size distribution in the *fog-2(q71)*; *msh-2*(RNAi) knockdown MA lines had a far greater proportion of 1-bp indels relative to the wildtype MA lines (Supplementary Figure S6, A and B; *t*-test: *t = *131.9, *P *<* *2.2 × 10^−16^). Single nucleotide indels comprise 97% (6,960/7,180) of all deletions and 95% (3,513/3,681) of all insertions, yielding a net loss of 3,447 bp. Indels >1-bp yield a further net loss of 381 bp. Across all 19 *fog-2(q71)*; *msh-2*(RNAi) knockdown MA lines, this deletion bias amounts to a net loss of 3,828 bp, an average of 201.5 bp per genome, and 5-bp per genome per generation. This net loss is ∼21× higher than the wildtype rate of 0.24 bp per genome per generation (Konrad *et al.* 2019).

### G/C homopolymeric runs are more prone to indel mutations than A/T runs

G/C homopolymeric runs incur, on average, more indels per bp than A/T homopolymeric runs (Supplementary Figure S7A). The frequency of homopolymeric A/T runs in the genome is significantly higher than those of G/C nucleotides (Supplementary Figure S7B), which explains the higher occurrence of A/T indels despite the greater propensity of G/C homopolymeric runs to gain or lose a base pair. Both, indel type (deletions *vs* insertions: three-way ANOVA: *F *=* *47.45, *P *=* *1.77 × 10^−9^) and base pair type (A/T *vs* G/C: three-way ANOVA: *F *=* *13.22, *P *=* *5.16 × 10^−4^) have a significant effect on the per base pair indel rates. G/C homopolymeric runs have significantly higher rates of deletions than A/T homopolymeric runs (Tukey’s Multiple Comparisons: *P* *= *0.009), while insertions do not differ significantly (Tukey’s Multiple Comparisons: *P* *= *0.24). Additionally, neither A/T nor G/C insertion and deletion rates are constant across homopolymeric runs of different lengths (Supplementary Figures S7, C and D). Shorter G/C runs display higher insertion rates whereas longer G/C runs exhibit higher deletion rates. Additionally, deletion rates increase with homopolymeric run lengths up to approximately 10 bp (G/C runs) and 11 bp (A/T runs). This heterogeneity in insertion and deletion rates in homopolymeric G/C runs results in a net gain of bases in runs <9 bp, and a net loss of bases in runs >9 bp (Supplementary Figure S7D).

### Dinucleotide microsatellites differ in indel dynamics from homopolymeric runs

Dinucleotide and polynucleotide repeats have, on average, slightly more insertions per nucleotide run than deletions (Supplementary Figure S7E). However, heterogeneity in the relative deletion and insertion rates between individual dinucleotide run types is apparent once the different dinucleotide runs are compared with one another (Supplementary Figure S7F). AC and AG dinucleotide runs experience slightly higher deletion rates than insertion rates, and CG/GC runs contained only deletions. In contrast, the insertion rate in AT/TA microsatellites was more than twofold higher than the deletion rate.

### *Mitochondrial mutations in fog-2(q71)*; *msh-2*(RNAi) knockdown MA lines

Five mitochondrial mutations were detected across the 19 *fog-2(q71)*; *msh-2*(RNAi) knockdown MA lines. All five mutations are heteroplasmic, although their respective intracellular frequencies ranged from relatively low (3.4–4.9%) to nearing fixation (81.7–93.7%) (Table  3). A nonsynonymous substitution in the *ND4L* gene resulting in a leucine to proline replacement had reached an approximately 94% frequency in MA line 16. Additionally, a deletion spanning 1,034 bp which removed the 3′ end of *COIII*, entire *tRNA-Thr* and the 5′ end of *ND4*, reached a frequency of ∼82% in MA line 16. Two frameshift mutations in the *ND5* gene occurred independently in two MA lines (∼5% and ∼4% frequency in MA line 4 and 34, respectively). Both of these frameshift mutations are single nucleotide insertions in the same homopolymeric run which we previously identified as a mutational hotspot for small indels in the *C. elegans* mitochondrial genome (Konrad *et al.* 2017). The number and intracellular frequencies of these mitochondrial mutations yield an overall mitochondrial mutation rate of 2.18 × 10^−7^/site/generation (95% CI: ±4.04 × 10^−7^ (Table  1). Although this estimated rate in the *fog-2(q71)*; *msh-2*(RNAi) knockdown MA lines is twofold greater than that calculated for wildtype MA lines (1.05 × 10^−7^/site/generation; Konrad *et al.* 2017), a meaningful statistical test between the two experiments is precluded owing to the presence of a large number of *fog-2(q71)*; *msh-2*(RNAi) knockdown MA lines with no mitochondrial mutations (Supplementary Figure S8A).

**Table 3:** List and details of five mtDNA variants identified in the *fog-2(q71)*; *msh-2*(RNAi) knockdown MA lines

MA line	MA generation	Position	Mutation	Effect	Gene(s)	Frequency	Context
16	33	721	T → C	Leu → Pro	*ND4L*	0.937	TCAAGAATCC[T]GGGTATGGTA
16	33	6,361–7,394	1034 bp del	Frameshift	*COIII, tRN A-Thr, ND4*	0.817	ATCATCTGGG[GTT…ATA]CATCTGGGAG
38	43	8,872	C → T	Pro → Leu	*COI*	0.034	GTATTTAATC[C]ACTTTTATTG
34	43	11,722	(T)_8_ → (T)_9_	Frameshift	*ND*5	0.040	ATTGGATTTG[T]_8_ATAGGTGGAA
4	44	11,722	(T)_8_ → (T)_9_	Frameshift	*ND5*	0.049	ATTGGATTTG[T]_8_ATAGGTGGAA

Five mutations were found in four lines, while the remaining 15 lines experienced no mutation. Only two of the five mutations approached fixation, while the remaining three were detected at low heteroplasmic frequencies. The mutations are displayed as changes on the major strand.

del, deletion

### *msh-2* knockdown does not affect the rates and length distributions of copy-number changes

Fifteen independent copy-number changes were detected across the 19 *fog-2(q71)*; *msh-2*(RNAi) knockdown MA lines (Table  4). A total of 16 *partial* and *complete* protein-coding genes were duplicated, yielding an overall gene duplication rate of 1.07 × 10^−6^ per gene per generation (95% CI: ± 8.36 × 10^−6^; Table  1). Six *partial* and *complete* protein-coding genes were deleted, yielding an overall gene deletion rate of 3.95 × 10^−7^ per gene per generation (95% CI: ± 3.71 × 10^−7^; Table  1). The gene duplication and deletion rates in the *fog-2(q71)*; *msh-2*(RNAi) knockdown MA lines are not significantly different from their counterparts in the wildtype MA lines (Konrad *et al.* 2018) (duplications: *t*-test: *t = *1.01, *P* *= *0.33; deletions: *t*-test: *t = *1.18, *P = *0.26) (Table  1). One of the copy-number variants (CNV) was a complex event comprising a coupled duplication and deletion event. Four of the 15 CNVs were detected on Chromosome V, which is consistent with previous work identifying chromosome V as the most CNV-prone chromosome in *C. elegans* (Maydan *et al.* 2007, 2010; Thompson *et al.* 2013; Konrad *et al.* 2018). Although the duplication and deletion rates calculated for the *fog-2(q71)*; *msh-2*(RNAi) knockdown MA lines appear to be somewhat lower than in wildtype MA lines, we did not test for significant differences between the two experiments due to the small number of *fog-2(q71)*; *msh-2*(RNAi) knockdown MA lines that contained copy-number changes. Only eight and four of our 19 *fog-2(q71)*; *msh-2*(RNAi) knockdown MA lines contained gene duplications and deletions, respectively.

**Table 4:** Summary of CNVs detected in the *fog-2(q71)*; *msh-2*(RNAi) knockdown MA lines

MA line	CNV type	Chr.	Start coordinate	Stop coordinate	Span (bp)	Coding (noncoding) complete genes	Coding (noncoding) partial genes
1	Deletion	II	4,667,216	4,669,226	2,011	0 (0)	2 (0)
1	Duplication	II	4,669,813	4,670,802	990	0 (0)	1 (0)
3	CNV: Gain	V	19,628,302	19,638,846	10,545	1 (1)	2 (0)
4	Deletion	II	13,063,885	13,064,154	270	0 (0)	1 (0)
6	Duplication	V	13,168,555	13,185,879	17,325	3 (1)	2 (1)
6	Duplication	X	1,509,086	1,526,337	17,252	1 (0)	0 (1)
7	Duplication	X	1,509,086	1,526,337	17,252	1 (0)	0 (0)
7	Deletion	V	5,261,877	5,262,010	134	0 (0)	1 (0)
19	Duplication	X	1,553,013	1,570,366	17,354	0 (1)	2 (0)
19	Duplication	IV	175,702	176,015	314	0 (1)	0 (0)
21	Duplication	IV	71,239	71,338	100	0 (0)	0 (0)
30	CNV: Gain	V	1,712,558	1,714,660	2,103	0 (0)	1 (1)
34	Duplication	X	1,509,086	1,526,109	17,024	1 (0)	0 (0)
38	Deletion	X	5,624,998	5,627,100	2,103	0 (0)	0 (0)
51	Duplication	III	12,152,320	12,157,380	5,061	0 (0)	1 (0)
66	Deletion	I	14,396,383	14,403,462	7,080	0 (0)	2 (0)

The majority of copy-number changes are either novel duplications or copy-number gains in preexisting multicopy regions. Only five deletions were detected, with smaller spans than the duplications. Consequently, not a single complete gene was deleted, while multiple complete genes were duplicated throughout the MA phase.

### *rDNA copy-number exhibits greater divergence in fog-2(q71)*; *msh-2*(RNAi) *knockdown MA lines*

Ribosomal RNA genes (18s, 28s, and 5.8s) are encoded in long tandem arrays at the end of Chromosome I in *C. elegans* (*C. elegans* Sequencing Consortium 1998). This region has previously been shown to exhibit extensive copy-number variation between nematodes, natural *C. elegans* isolates, and experimental MA lines (Bik *et al.* 2013; Thompson *et al.* 2013; Konrad *et al.* 2018). There is considerable variation in the estimated number of rRNA genes between the *fog-2(q71)*; *msh-2*(RNAi) knockdown MA lines, ranging from ∼72 to 207 copies. The *fog-2(q71)*; *msh-2*(RNAi) knockdown MA lines harbor significantly higher rDNA copy-number than the wildtype MA lines (Supplementary Figure S8B; *t = *3.26, *P = *0.0025). However, the variation in rDNA copy-number after the conclusion of each experiment did not differ significantly between the *fog-2(q71)*; *msh-2*(RNAi) knockdown and wildtype MA lines (*F *=* *1.04, *P* *= *0.99). The wildtype MA lines were propagated for up to 409 generations, with an average of 361 generations per line, whereas the *fog-2(q71)*; *msh-2*(RNAi) knockdown MA lines were maintained for up to 50 generations with an average of 40.3 generations per line. When the number of generations in each experiment is taken into account, there is significantly greater divergence of rDNA copy-number per generation in the *fog-2(q71)*; *msh-2*(RNAi) knockdown relative to wildtype MA lines (Supplementary Figure S8C; *F *=* *70.6, *P *=* *1.34 × 10^−11^). This difference could be the result of higher recombination rates within the rDNA gene arrays of the *fog-2(q71)*; *msh-2*(RNAi) knockdown MA lines. Alternatively, the difference could be the result of higher ancestral rDNA copy-number in *fog-2(q71)*; *msh-2*(RNAi) knockdown MA lines compared with their wildtype counterparts, and hence a higher probability or opportunity for unequal recombination to occur. The average copy-number change relative to the ancestral state was significantly different between the wildtype and *fog-2(q71)*; *msh-2*(RNAi) knockdown MA lines (*t*-test: *t = *2.57, *P* *= *0.015); the latter had an average loss of seven copies of rDNA per line, which was not significantly different from a zero net loss (Supplementary Figure S8C; *t*-test: *t = *0.95, *P* *= *0.35). This stands in contrast to previous results from wildtype MA lines which had an average increase of 20 rDNA copies per line (Konrad *et al.* 2018) (Supplementary Figure S8B; *t*-test: *t = *2.62, *P* *= *0.02).

## Discussion

DNA repair systems contribute to the evolution of genomes in multiple ways. The reduction in mutation rate by DNA repair limits genome degradation and yet, at the same time, can limit the supply of potentially beneficial mutations. The specificity of repair systems contributes to the evolution of base composition as well as the prevalence and distribution of genomic features such as the length and types of DNA sequence repeats. The efficiency and specificity of DNA repair can be constrained by the chemical and spatial configuration of mismatches in the double helix as some mismatches are more easily recognized than others. However, DNA repair could also adapt to the types and frequency of mutations in the unrepaired DNA. For instance, repair systems could adapt to preferentially repair mismatches that are particularly frequent in a genome at the expense of mismatches that are rare, or they could adapt to repair mutations that are more likely to be harmful, such as frameshifts or transversions. The specificity of DNA repair in a species would then be a function of the tradeoffs between structural constraints, the fitness cost of spontaneous mutations in the absence of repair and the cost of repair itself. Finally, the effective population size of species can set limits to adaptation of DNA repair systems. The analyses of mutation rates in DNA repair-deficient organisms increases our understanding of how spontaneous mutations arise and helps identifying DNA sequence features that are more or less susceptible to mutations. Furthermore, a comparative analysis of the mutation spectrum in repair-proficient and -deficient organisms contributes to our understanding of the DNA repair systems themselves and their evolution.

This study provides an evolutionary genome-wide view of the effect of MMR impairment on the mutational landscape in *C. elegans* by severely limiting the assembly of the *mutSα* complex through RNA-interference of the *msh-2* gene in obligately outcrossing *fog-2(q71)*; *msh-2*(RNAi) knockdown MA lines. This combination of (1) the near elimination of natural selection on spontaneous mutations, and (2) knockdown of a key MMR gene permits a more refined understanding of the raw rate and spectrum of mutational input prior to MMR. We compared the mutation rates and spectra in this set of MMR-impaired obligately outcrossing *C. elegans* MA lines to those observed in another spontaneous MA experiment with the wildtype N2 selfing strain of *C. elegans* (Konrad *et al.* 2017, 2018, 2019). As far as we know, there is no evidence yet to suggest that mutation rates and spectra differ between self-fertilizing *C. elegans* and the outcrossing populations employed in this study. However, this does not rule out that obligately outcrossing and selfing strains of *C. elegans* may differ with respect to mutation rates and DNA repair. Indeed, mutation rates and repair may differ between *C. elegans* males and hermaphrodites/females which we test for in this study by comparing the accumulation of mutations on autosomes *vs* the X chromosome. Several additional caveats are in order. Exposure to different *E. coli* strains as a food source can result in *C. elegans* exhibiting (1) different phenotypes with respect to development, reproduction and metabolism, as well as (2) unique transcriptional responses (Neve *et al.* 2020; Stuhr and Curran 2020). Although there is no evidence in both studies to suggest that our use of *E. coli* HT115 could alter the rate or spectrum of spontaneous mutations or the ability of the MMR machinery to repair mutations, it is conceivable that these environmentally induced differences may also influence the mutation rate.

### *msh-2* knockdown in outcrossing *C. elegans* lines is effective and increases the genome-wide nuclear mutation rate by more than 100-fold

RNAi-induced *msh-2* knockdown in obligately outcrossing *C. elegans* MA lines comprising this study resulted in a nuclear mutation rate of 2.65 × 10^−7^/site/generation (SNPs and small indels), representing a 105× increase over the comparable mutation rate of 2.52 × 10^−9^/site/generation in the *C. elegans* wildtype (selfing), MMR-proficient genetic background (Konrad *et al.* 2019). The rates of synonymous and nonsynonymous mutations were not significantly different from each other in our *fog-2(q71)*; *msh-2*(RNAi) knockdown MA lines, which is consistent with negligible purifying selection. At the genic level, frameshift mutations resulting from small indels were 50-fold higher than in our previous experiment with wildtype N2 worms, and nonsynonymous mutations were 24-fold higher. A comparison of prior analyses of wildtype MA lines using Sanger sequencing of ∼20 kb (0.02%) of the *C. elegans* genome suggested a ∼48× increase in mutation rates in *msh-2* and *msh-6* knockout lines of *C. elegans* (Denver *et al.* 2005, 2006). Denver *et al.* (2006) additionally conducted a partial genomic study of two additional excision repair pathways (BER and NER) in *C. elegans* which exhibited a 17–28× increase in overall mutation rates, suggesting that the MMR pathway plays the lead role in minimizing the mutation load in *C. elegans*.

The nuclear base substitution rate of 4.22 × 10^−8^/site/generation of the *fog-2(q71)*; *msh-2*(RNAi) knockdown MA lines was ∼23× greater than that observed for wildtype MA lines by Konrad *et al.* (2019). As is the case for *mutS* heterodimers, only one *mutL* complex, MutLα, encoded by two *mutL* homologs *mlh-1* and *pms-1*, is present in *C. elegans*. Hence, defects in MutLα function are expected to lead to similar mutational spectra as those in the MutSα complex. However, *mlh-1*Δ and *pms-1*Δ knockout mutants exhibited substantially greater increase in the base substitution rate (∼75×; Meier *et al.* 2018) compared with the ∼23× increase observed by us under *msh-2* knockdown. The most striking difference in mutation rate between the *C. elegans msh-2* knockdown MA lines in this study relative to wildtype MA lines (Konrad *et al.* 2019) was the ∼328× increase in small indels (Figure  1). The knockout of *mutL* homologs *mlh-*1 and *pms-1* in *C. elegans* each resulted in ∼440× increase in the small indel rate (Meier *et al.* 2018). One possibility for the differential increase in base substitution and indel rates under *mutL-* (Meier *et al.* 2018) *vs* *mutS*-impairment (this study) is that defects in these different components of MMR have different consequences for mutation and repair. Alternatively, RNAi-induced knockdown in our study did not fully inactivate *msh-2*. However, differences in mutation rate between *mutS and mutL* lines in the same species have been noted before (Lee *et al.* 2012; Long *et al.* 2016).

### Interspecific variation in the efficiency and specificity of MMR systems

**Figure 7:**
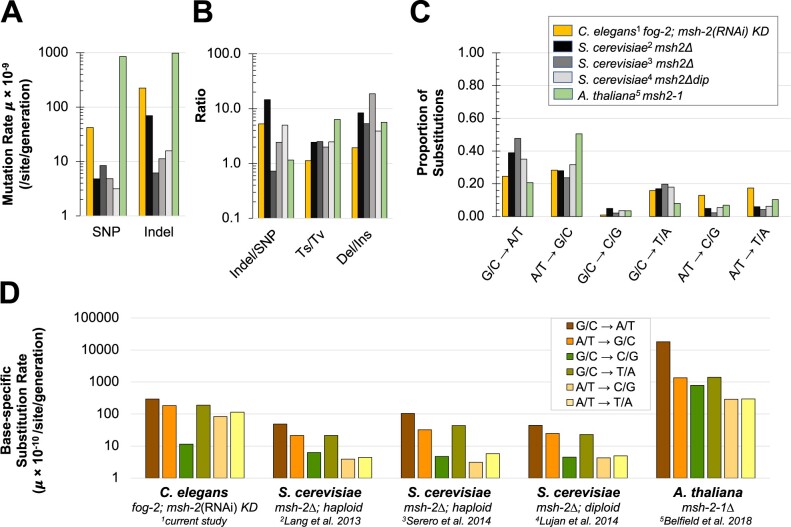
Comparisons of mutation rate and spectrum in MMR-deficient lines of several model eukaryotic species. The mutational spectrum of the *Caenorhabditis elegans* obligately outcrossing *fog-2(q71)*; *msh-2*(RNAi) knockdown MA lines analyzed in the present study^1^ are compared with knockout mutations of either *msh-2* or *msh-6* of the MutS*α* heterodimer in haploid (^2^Lang *et al.* 2013; ^3^Serero *et al.* 2014) and diploid *Saccharomyces cerevisiae* (^4^Lujan *et al.* 2014), and *Arabidopsis thaliana* (^5^Belfield *et al.* 2018). (A) SNP and indel rates estimated for the different datasets span three orders of magnitude. Substitution and indel rates in *A. thaliana* are approximately equal, while yeast species (except ^3^Serero *et al.* 2014) and the obligately outcrossing strain of *C. elegans* show markedly lower substitution rates than their respective indel rates. (B) The number of indels per SNP varies widely between taxa and between experiments with *Saccharomyces*. Transition/transversion (Ts/Tv) ratios are highest in *A. thaliana* and lowest for the obligately outcrossing strain of *C. elegans*. Deletions outnumber insertions in all of these species and the deletion/insertion ratio is lowest in *C. elegans*. (C) The mutational spectrum differs between species. *A. thaliana* has a greater share of G/C → A/T transitions and lower share of A/T → G/C transitions relative to the other species. Obligately outcrossing *C. elegans* has a greater share of A/T → C/G or T/A transversions relative to other species. (D) The base-specific substitution rates vary widely between different taxa.

Genome-wide mutation rates (base substitutions and indels) in an MMR-deficient background can be quite variable across as well as within species, reflecting both species-specific differences in the efficiency and specificity of MMR and the influence of genetic background within a species, in addition to methodological differences in the analysis of mutations (Long *et al.* 2018, Supplementary Tables S3; Figure  7). MMR-deficiency increases the genome-wide mutation rate by one to three orders of magnitude. We further compare mutation rates under *msh-2* impairment or deficiency in an MA setting in two other eukaryotes namely *S. cerevisiae* and *A. thaliana.* The increase in base substitution rates under *msh-2* deficiency in outcrossing *C. elegans* (23×; this study) is more similar to *S. cerevisiae* (15–27×; Lang *et al.* 2013; Lujan *et al*. 2014; Serero *et al.* 2014) than to the other multicellular eukaryote *A. thaliana* (170×; Belfield *et al.* 2018).

The relative increase in small indels under *msh-2* deficiency in outcrossing *C. elegans* (328×; this study) is much lower relative to *S. cerevisiae* (703–3500×; Lang *et al.* 2013; Serero *et al.* 2014) and *A. thaliana* (1000×; Belfield *et al.* 2018). Irrespective, the three eukaryotic species exhibit two to three orders of magnitude increase in small indel rates, suggesting that the MMR system in eukaryotes is far more efficient in the repair of small indels relative to base substitutions. The consequences of mismatch-repair deficiency for indels relative to base substitutions vary greatly between taxa (Supplementary Table S3). In the six species of prokaryotes thus far studied, MMR-defective strains for four species (*Vibrio* *fischeri*, *B. subtilis*, *P. fluorescens*, and *E. coli*) had ratios of fold-change increases in the small indel rate relative to the base substitution rate ranging from 0.32 to ∼1.0, revealing either (1) a base substitution-bias or (2) approximately equal rates of indels and base substitutions under MMR-deficiency. Two prokaryotic species, *Vibrio* *cholerae* and *Deinococcus* *radiodurans*, however, had ratios of increases in the small indel rate relative to the base substitution rate ranging from 1.67 to 5.25 signifying a small indel bias relative to base substitutions under MMR-deficiency. *Caenorhabditis* *elegans* shares similarities with the other two eukaryotes, *A. thaliana* (ratio of ∼5; calculated from Belfield *et al.* 2018) and *S. cerevisiae* (ratios of ∼233 calculated from Lang *et al.* 2013, and ∼31 calculated from Serero *et al.* 2014) which also display a far greater increase in small indels relative to base substitutions in an MMR-impaired background. In eukaryotes, MMR-deficiency appears to lead to a far greater increase in the number of indels relative to base substitutions, suggesting a functional divergence in the MMR machinery of eukaryotes and prokaryotes. However, elucidating the causes of variation in the specificity of MMR requires a far broader taxonomic sample of studies investigating the consequences of mismatch-repair deficiency on the mutational spectrum and fitness.

### Dependence of small indel rate on base composition and length of homopolymeric run

In our study, while the overall indel mutation rate increased ∼328×, single base pair insertion and deletion rates increased by 638× and 517×, respectively. These 1 bp indels were predominantly found in homopolymeric runs. The shortest indels (1–2 bp in length) are both (1) most common and (2) exhibit the greatest increase in mutation rates in our *fog-2(q71)*; *msh-2*(RNAi) knockdown MA lines compared with wildtype MA (Konrad *et al.* 2019)*.* The preference for repairing the shortest indels by MMR has also been observed in prokaryotes (Long *et al.* 2018). The average increase in the insertion rate during *msh-2* knockdown compared with wildtype lines was significantly higher (422×) than that for deletions (288×). Although both the wildtype and *fog-2(q71)*; *msh-2*(RNAi) knockdown MA lines exhibited a deletion bias, it was significantly lower in the latter. These results suggest that MMR in *C. elegans* is better at detecting or repairing small insertions relative to deletions.

It is well-established that runs of identical nucleotides are mutational hotspots for small indels (Streisinger *et al.* 1966; Lee *et al.* 2012). Runs of G/C were significantly less stable than those of A/T in our *fog-2(q71)*; *msh-2*(RNAi) knockdown MA lines, especially with respect to deletions. Higher indel rates in homopolymeric runs of G/C than A/T have been previously observed in a partial genomic analysis (∼20 kb) in *C. elegans* (Denver *et al.* 2005) and in *S. cerevisiae* MMR-deficient lines (Gragg *et al.* 2002; Lang *et al.* 2013). Additional differences between G/C and A/T homopolymeric runs in our *C. elegans* MMR-impaired lines include the tendency for an insertion-bias in shorter (<9 bp) G/C runs *vs* the tendency toward a deletion-bias in A/T runs, regardless of the length of the run. MMR-deficient lines of *E. coli* also exhibit an insertion-bias in G/C homopolymeric runs (Lee *et al.* 2012).

Lee *et al.* (2012), among others, have previously proposed that the indel rate in homopolymeric runs is dependent on the length of the run. The indel rates in homopolymeric runs in *E. coli mutL*, *V.* *fischeri mutS*, and *V. cholerae mutS*, increase with the length of a run of up to 10 bp (Lee *et al.* 2012; Dillon *et al.* 2017). However, the relationships between indel rates and the length of the homopolymeric runs in bacteria may have been limited by a small number of runs with >10 bp. In *C. elegans*, the indel rate peaks in 11 bp runs and is lower in runs that were either shorter or longer, both in wildtype MA lines (Konrad *et al.* 2019) and *fog-2(q71)*; *msh-2*(RNAi) knockdown MA lines comprising this study. However, when the indel frequency was analyzed separately in G/C and A/T runs in wildtype and *fog-2(q71)*; *msh-2*(RNAi) knockdown MA lines of *C. elegans*, the A/T runs followed this same pattern whereas the G/C indel rates peaked in runs of 8 bp. Interestingly, the indel rates in homopolymers in *A. thaliana*, which were primarily A/T, also peaked at 11 bp (Belfield *et al.* 2018). The fact that we see the same relationship between indel rates and the length of A/T homopolymeric runs in *C. elegans* and *A. thaliana* could be a mere coincidence. However, it merits further investigation into possible general rules of short indel dynamics and their consequences for genome evolution.

### Dinucleotide microsatellites differ in indel dynamics from homopolymeric runs

In contrast to homopolymeric repeats, dinucleotide and polynucleotide repeats had an average tendency for more insertions than deletions in our outcrossing MMR-impaired MA lines. However, this tendency appeared to vary based on the specific composition of the repeat unit. For example, (AT)_*n*_ dinucleotide repeats appeared to have 2× more insertions than deletions, a mutational bias that has also been observed in a genome-wide analysis of *S. cerevisiae* MMR-deficient lines (Lang *et al.* 2013). In contrast to (AT)_*n*_ dinucleotides, (AC/GT)_*n*_, and (AG/CT)_*n*_ dinucleotide repeats in our MMR-deficient lines appear to have marginally higher deletion rates whereas only deletions were observed in (GC)_*n*_ repeats. Lang *et al.* (2013) also observed a slight deletion bias, albeit nonsignificant, in (AC/GT)_*n*_ repeats in yeast. Degtyareva *et al.* (2002) analyzed five microsatellite loci [(GT)_14_, (GT)_26_, (GT)_59_, (AAT)_28_, and (AAAT)_43_] in selfing *C. elegans msh-2* deficient lines and observed a 2.2-fold higher incidence of insertions to deletions (33 *vs* 15) in the pooled sample. The discrepancy between our results and those of Degtyareva *et al.* (2002) may stem from (1) limited sampling in the latter study as compared with a genome-wide analysis, or (2) due to different repair efficiency in knockout *vs* knockdown experiments, or (3) mode of reproduction (selfing *vs* outcrossing).

### *msh-2* knockdown leads to increased transition bias

MMR repairs transitions more efficiently than transversions, which results in an increased transition bias in MMR-impaired MA lines. The Ts/Tv was significantly higher in the *fog-2(q71)*; *msh-2*(RNAi) knockdown MA lines relative to the wildtype MA lines (Konrad *et al.* 2019), increasing from 0.67 to 1.12, or 1.7×. In *A. thaliana*, the Ts/Tv ratio increased 2.8× in *msh-2* MA lines (Belfield *et al..* 2018). The transition bias also increased in *S. cerevisiae mutS* deletions (Lang *et al.* 2013; Serero *et al.* 2014). In bacteria, the increase in the Ts/Tv ratio in MMR-deficient strains compared with wildtype ranges from 3× to 48× (Long *et al.* 2018, and references therein).

In MA experiments with eukaryotes, G/C → A/T transitions are the primary contributor to an increased Ts/Tv bias in MMR-deficient lines (Lang *et al.* 2013; Serero *et al.* 2014; Belfield *et al.* 2018). The disproportionately high share of G/C → A/T transitions of all spontaneous base substitutions have been explained by deamination of methylated cytosines (Lutsenko and Bhagwat 1999). Both *Saccharomyces* and *Arabidopsis* employ CpG methylation as a part of their epigenetic toolkit which is consistent with the differences in G/C → A/T transitions between these two species (Finnegan *et al.* 1996; Tang *et al.* 2012); *S. cerevisiae* has relatively low levels of CpG methylation (Tang *et al.* 2012), and exhibits a much lower contribution of G/C → A/T transitions to the mutational spectrum than *A. thaliana*, which has higher levels of CpG methylation (Takuno *et al.* 2016). In *C. elegans*, however, the relative contribution of G/C → A/T transitions to the substitution spectrum is much lower (25%) compared with *S. cerevisiae* (35–48%) or *A. thaliana* (76%) despite similar genomic G + C-content (38% in *S. cerevisiae* and 36% in *C. elegans*, and *A. thaliana*) (Lang *et al.* 2013; Serero *et al.* 2014; Belfield *et al.* 2018). The difference in the rates of transitions in *C. elegans* relative to other eukaryotes could be due to the lack of the CpG methylation (Simpson *et al.* 1986). Interestingly, *S. pombe*, which is also devoid of CpG methylation (Antequera *et al.* 1984) shows a similarly low contribution of G/C → A/T transitions to the mutation spectrum as *C. elegans* (Sun *et al.* 2016). This further implicates differences in CpG methylation in driving the variation between species in G/C → A/T transitions, and the transition bias.

The variation in the efficiency of repairing different mismatches also influences the evolution of genome base composition. In *C. elegans*, the mutation bias toward a lower GC-content is greater in the wildtype MA lines relative to their *fog-2(q71)*; *msh-2*(RNAi) knockdown counterparts. A/T → G/C transitions were repaired more efficiently than G/C → A/T transitions and A/T → C/G transversions were repaired more efficiently than G/C → T/A transversions (Figure  2B). Hence, the MMR system appears to contribute to the mutational bias toward a greater AT-content in *C. elegans*.

### Local sequence-context influences mutation rates in outcrossing MMR-deficient lines

The DNA sequence flanking mutated bases appears to influence the probability of mutation in the *msh-2* MA lines. Both A/T and G/C base pairs are most prone to mutations when they are flanked by a 5′-G and a 3′-C. Several empirical studies of the local sequence-context on mutation rate in bacteria have found that guanines and cytosines flanking the focal nucleotide increase the probability of a mutation (Lee *et al.* 2012; Long *et al*. 2015; Sung *et al.* 2015; Takemoto *et al.* 2018). The association between flanking G + C-content and mutation rate implicates strong base pairing of flanking nucleotides in stabilizing mismatched bases (Sung *et al.* 2015).

Even more striking is the context-dependence of A/T → T/A transversions, which were more common when the mutated A/T base pair was flanked by a 5′-A and a 3′-T than when flanked by other bases. A/T → T/A transversions also exhibited context-dependence in the wildtype MA lines, but with a higher mutation rate when flanked by 5′-T and a 3′-A (Konrad *et al.* 2019; Saxena *et al.* 2019). In our *fog-2(q71)*; *msh-2*(RNAi) knockdown MA lines, A/T → T/A transversions were particularly frequent within or adjacent to repetitive sequences, especially at the boundaries of homopolymeric runs.

### Significant variation in rDNA copy-number

In contrast to duplications and deletions of single-copy genes, the dynamics of rDNA copy-number are different between the *fog-2(q71)*; *msh-2*(RNAi) knockdown and wildtype MA lines. Three independent experiments have found that rDNA copy-number increased during MA in wildtype *C. elegans* lines (Bik *et al.* 2013; Konrad *et al.* 2018; Wahba *et al.* 2021). Although our *fog-2(q71)*; *msh-2*(RNAi) knockdown MA lines displayed a significant variation in rDNA copy-number, there was no significant change in the average copy-number. Furthermore, there was a significant difference in the direction of change in rDNA copy-number between this study and our *C. elegans* wildtype MA experiment of Konrad *et al.* (2018). Could *msh-2* be involved in rDNA copy-number control and bias copy-number changes toward an increase rather than a decrease or random directional change? There are additional considerations that could potentially explain the difference between our outcrossing *msh-2* knockdown and wildtype MA results. First, the ancestral rDNA copy-number differed between the two experiments. The N2 ancestor of the wildtype MA lines was estimated to have 98 copies (Konrad *et al.* 2018) whereas the *fog-2(q71)* ancestor of the *msh-2* knockdown MA lines contained an estimated 160 copies of rDNA. The range in rDNA copy-number may be limited by lower and upper boundaries, and because the N2 ancestor of the wildtype MA lines had fewer copies, a reduction in rDNA copy-number may more frequently fall below what is permissible without a significant reduction in fitness. Consequently, purifying selection might operate more often on rDNA copy-number losses than increases in the wildtype MA lines. This would result in the wildtype MA lines displaying evidence of copy-number increase more frequently than decrease and give the impression of a mutational bias toward a copy-number increase. Furthermore, the greater ancestral copy-number of rDNA in the *fog-2(q71)*; *msh-2*(RNAi) knockdown MA lines may simply provide more substrate for generating new copy-number variation through recombination. This in turn would lead to more copy-number changes per generation regardless of the functionality of *msh-2*. Recently, variation in rDNA copy-number has been associated with broad changes in gene expression in *Drosophila* and humans (Paredes and Maggert 2009; Gibbons *et al.* 2014). Further experiments are clearly needed to understand the evolutionary dynamics, and the transcriptional and phenotypic consequence of rDNA copy-number dynamics in *C. elegans.*

### The X-chromosome has lower substitution rate and higher indel rate than the autosomes

Male-biased mutation rates have been observed in many species of mammals, bird, and plants (Whittle and Johnston 2003; Wilson Sayres and Makova 2011; Jónsson *et al.* 2017). *Caenorhabditis* *elegans* has an XO sex-determination system wherein wildtype worms are predominantly self-fertilizing XX hermaphrodites with XO males in rare frequency. However, because the *msh-2* MA experiments were performed in a *fog-2* genetic background which results in obligate outcrossing and equal numbers of males and females, two-third of the X chromosomes are in females and one-third in males in each generation. This predicts a lower observed mutation rate on the X chromosome than on the autosomes if males have a higher mutation rate than females. For example, MA experiments with *Drosophila* have detected slightly higher mutation rates in males due to the X chromosome having lower mutation rate than the autosomes, although the difference was not statistically significant (Keightley *et al.* 2009). Interestingly, base substitution rates on the X are significantly lower than on the autosomes in the *C. elegans fog-2(q71)*; *msh-2*(RNAi) knockdown MA lines, which is consistent with a male-biased mutation rate. In contrast, we found higher small indel rates on the X chromosome, consistent with a female-bias in indel rates. Differences in mutation rates between the sexes in these experiments might, in principle, result from differences between males and females in their sensitivity to *msh-2* knockdown by RNAi, although the fact that the direction of change is different for substitutions and indels makes this possibility less likely. However, chromosome location was a poor predictor of mutation rate in the logistic regression analysis and there did not appear to be an X chromosome effect. Although the differences in average mutation rate between the X chromosome and the autosomes are consistent with sex-specific differences in mutation rate, we cannot rule out the influence of chromosomal variation with respect to the number and distribution of mutable sites. In addition to differences between the X chromosome and the autosomes, other aspects of genomic location and sequence-context were associated with the mutation rate. For example, both base substitutions and indels were less likely to occur in exons than in intergenic regions, and less likely in chromosomal cores *vs* arms. However, as with the X chromosome to autosome comparison, recombinational domains (cores, arms, and tips) and location in coding *vs* noncoding DNA were poor predictors of mutability. Most of the variation in mutation rate within the genome could be explained by sequence complexity, sequence repeats, and G + C-content. Furthermore, the indels were dominated by 1 bp deletions and insertions in runs of As and Ts, and the nonrandom distribution of A/T homopolymeric runs across the genome appears to explain the variation in indel rates.

In summary, this study combines an MA experimental framework coupled with WGS to investigate alterations to the mutation rate and spectrum under impaired functionality of the MutS homolog, *msh-2* in outcrossing *C. elegans* lines, at both the mitochondrial and nuclear levels. The large number of mutations, 13,986 in total, enabled the most comprehensive view of the characteristics and distribution of mutations in any given *C. elegans* genotype. Some results were surprising, such as the great rate of divergence in rRNA copy-number compared with our previous study of wildtype *C. elegans* lines, and differences in mutation rates between the X chromosome and autosomes, which is consistent with sex-specific mutation rates. These differences in *C. elegans* may be a consequence of a nonrandom distribution of mutable sites in the genome, which also affects differences between coding and noncoding DNA and between chromosome arms and cores. However, the results suggest that possible sex-specific differences in mutation rates in *Caenorhabditis* should be further investigated. The probability of mutation is influenced by several factors that are shared with other species, such as sequence complexity and flanking nucleotide G + C-content. In contrast, the sequence context of A/T to T/A transversions at the ends of homopolymeric runs have not been readily apparent in other species. MMR impairment has variable consequences in different species, which demonstrates that although the MMR system is evolutionarily conserved, the specificity of MMR in different taxa can vary and is evolutionary labile. Whether the specificity of MMR is any given species is adapted to the distribution and fitness effects of endogenous DNA replication errors in those species is an open question.

## Data availability

Whole-genome sequence data of all experimental lines and the ancestral control have been deposited at the National Center for Biotechnology Information Sequence Read Archive (Bioproject PRJNA 554105).

Supplementary material is available at *G3* online.

## Supplementary Material

jkab364_Supplementary_Figures_Tables

jkab364_Supplementary_Data
