# Supplementary material for: Mutation rate and spectrum in obligately outcrossing Caenorhabditis elegans mutation accumulation lines subjected to RNAi-induced knockdown of the mismatch repair gene msh-2
Source: G3 (Bethesda). 2021 Oct 21;12(1):jkab364. doi: 10.1093/g3journal/jkab364 (PMC8727991; doi:10.1093/g3journal/jkab364)
Supplement: jkab364_Supplementary_Figures_Tables [file jkab364_supplementary_figures_tables.pdf]

Supplemental Material

Katju ***et al. "Mutation spectrum in obligately outcrossing mutation accumulation lines of Caenorhabditis elegans subjected to RNAi-induced knockdown of the mismatch repair gene msh-2."***

Supplemental Table S1. Summary of obligately outcrossing *fog-2(q71)*; *msh-2*(RNAi) MA lines, generations propagated under population size bottlenecks under a mutation accumulation (MA) experimental regime, and sequence coverage per genome.

Genotype	MA Line	MA Generations	Coverage
fog-2(q71); msh-2 (RNAi)	1	38	18
fog-2(q71); msh-2 (RNAi)	3	44	17
fog-2(q71); msh-2 (RNAi)	4	44	15
fog-2(q71); msh-2 (RNAi)	6	33	16
fog-2(q71); msh-2 (RNAi)	7	28	17
fog-2(q71); msh-2 (RNAi)	16	33	17
fog-2(q71); msh-2 (RNAi)	19	43	18
fog-2(q71); msh-2 (RNAi)	21	39	17
fog-2(q71); msh-2 (RNAi)	28	36	19
fog-2(q71); msh-2 (RNAi)	30	42	16
fog-2(q71); msh-2 (RNAi)	34	43	16
fog-2(q71); msh-2 (RNAi)	35	43	18
fog-2(q71); msh-2 (RNAi)	38	43	16
fog-2(q71); msh-2 (RNAi)	45	41	16
fog-2(q71); msh-2 (RNAi)	50	44	16
fog-2(q71); msh-2 (RNAi)	51	45	16
fog-2(q71); msh-2 (RNAi)	66	45	16
fog-2(q71); msh-2 (RNAi)	68	39	16
fog-2(q71); msh-2 (RNAi)	74	43	14
Ancestral fog-2(q71) pre-MA control	PMAC	0	30

Supplemental Table S2. Fitted coefficients and Odds Ratios *(OR)* for predictor contribution to site mutability using a logistic regression approach.

Predictor	Coefficient	Odds Ratio
V	0.24	1.27
IV	0.04	1.04
I	0.00	1.00
X	0.00	1.00
II	-0.05	0.95
III	-0.17	0.84
Intron	0.01	1.01
3'UTR	0.00	1.00
5'UTR	0.00	1.00
Intergenic	-0.01	0.99
Exon	-0.07	0.93
Core	0.00	1.00
Tip	-0.01	0.99
Arm	-0.04	0.96
Recombination Rate	0.03	1.03
Germline Expressed	0.00	1.00
Repeat Sequence	1.49	4.42
Seq. Complexity (40 bps)	0.33	1.39
CG Content (40 bps)	-0.13	0.88
GCC/GGC	1.59	4.90
CGC/GCG	1.42	4.12
AGC/GCT	1.33	3.79
AGG/CCT	1.09	2.97
GAC/GTC	0.91	2.49
AAT/ATT	0.80	2.22
ACG/CGT	0.76	2.15
CAC/GTG	0.75	2.12
GCA/TGC	0.75	2.11
AAC/GTT	0.44	1.56
CTC/GAG	0.40	1.50
CAG/CTG	0.40	1.49
AAG/CTT	0.38	1.46
ATC/GAT	0.28	1.33
ACC/GGT	0.26	1.30
ATG/CAT	0.23	1.26
ACT/AGT	0.23	1.26
CCC/GGG	0.19	1.21
GTA/TAC	0.17	1.19
ACA/TGT	0.13	1.14
CCG/CGG	0.13	1.13
GGA/TCC	0.08	1.08
AGA/TCT	0.01	1.01
CGA/TCG	-0.08	0.92
CTA/TAG	-0.08	0.92
TAA/TTA	-0.23	0.80
CCA/TGG	-0.40	0.67
GAA/TTC	-0.41	0.67
ATA/TAT	-0.52	0.60
TCA/TGA	-0.71	0.49
CAA/TTG	-0.73	0.48
AAA/TTT	-1.64	0.19

Supplemental Table S3. Summary of fold-change in the nuclear base substitution and small indel rates in MMR-deficient MA lines of various prokaryote and eukaryote species relative to their MMR-proficient counterparts. μ_{indel} and μ_{BS} refer to the mutation rates for nuclear small indels and base substitutions, respectively.

**Species**	**Fold-change in μ_{indel}**	**Fold-change in μ_{BS}**	**Fold-change μ_{indel} / Fold-change μ_{BS}**	**Reference**
*Vibrio fischeri mutS*Δ	102	317	0.32	Dillon *et al.* 2017
*Bacillus subtilis mutS*Δ	44	101	0.44	Sung *et al.* 2015
*Pseudomonas fluorescens mutL*Δ	176	279	0.63	Long *et al.* 2018
*Pseudomonas fluorescens mutS*Δ	206	309	0.67	Long *et al.* 2018
*Escherichia coli mutL*Δ	136	141	0.96	Lee *et al.* 2012
*Escherichia coli mutS*Δ	119	115	1.03	Long *et al.* 2016
*Vibrio cholerae mutS*Δ	142	85	1.67	Dillon *et al.* 2017
*Deinococcus radiodurans mutS1*Δ	15	4	3.75	Long *et al.* 2018
*Deinococcus radiodurans mutL*Δ	21	4	5.25	Long *et al.* 2018
*Saccharomyces cerevisiae msh-2*Δ	703	23	30.57	Serero *et al.* 2014
[a]*Saccharomyces cerevisiae msh-2*Δ	3500	15	233.33	Lang *et al.* 2013
Caenorhabditis elegans fog-2(q71); msh-2(RNAi)	328	23	14.26	This study
*Arabidopsis thaliana msh2-1*Δ	1000	170	5.88	Belfield *et al.* 2018

[a] fold-changes in base substitution and indel rates under MMR-deficiency were generated by comparing to wildtype (MMR-proficient) rates calculated by Zhu *et al.* (2014)

Supplemental Table S4. Summary of fold-change in the nuclear base substitution rates in MMR-deficient MA lines of three eukaryote species relative to their MMR-proficient counterparts.

**Substitution type**	**Fold-increase in *C. elegans* under *msh-2 KD***	**Fold-increase in *A. thaliana* under *msh-2 KO***	**Fold-increase in *S. cerevisiae* under *msh-2 KO***
G/C → A/T	22	190	47
A/T → G/C	54	115	51
G/C → C/G	3	68	10
G/C → T/A	26	101	24
A/T → C/G	46	56	9
A/T → T/A	13	57	13

[a] fold-changes in base substitution rates under MMR-deficiency in obligately outcrossing *C. elegans* (this study) were generated by comparing to wildtype MMR-proficient rates calculated by Konrad *et al.* (2019)

[b] fold-changes in base substitution rates under MMR-deficiency in *A. thaliana* (Belfield *et al.* 2018) were generated by comparing to wildtype (MMR-proficient) rates calculated by Ossowski *et al.* (2010)

[c] fold-changes in base substitution rates under MMR-deficiency relative to wildtype in *S. cerevisiae* (Lujan *et al.* 2014)

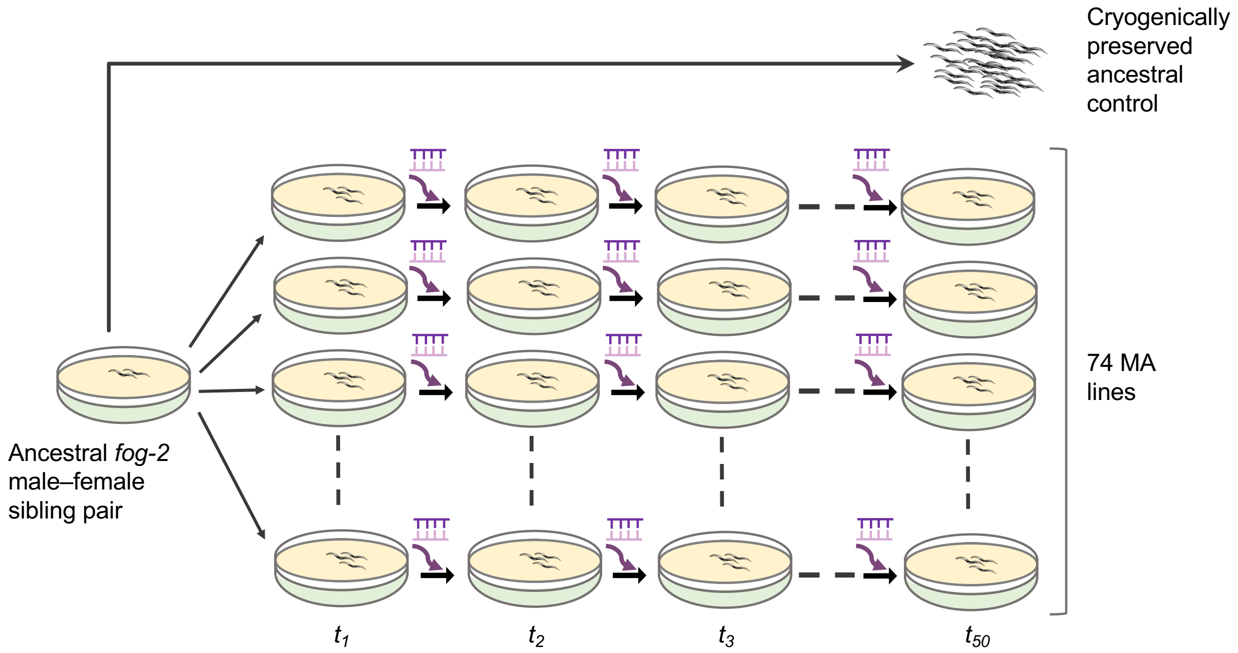

Supplemental Figure S1 Schematic of the *msh-2* knockdown mutation accumulation (MA) experiment in an obligately outcrossing strain of *C. elegans*. The MA experiment was established from a single male-female pair of an obligatory outcrossing *fog-2*(*q71lf*) mutant strain of *C. elegans*. A trio of one virgin female and two males from the descendants of the founding pair were each used to establish 74 MA lines. The accumulation of mutations was accelerated by simultaneously (i) bottlenecking populations ($N = 3$ individuals), and (ii) RNAi-induced knockdown of MMR gene *msh-2* (symbolized by the purple RNA-RNA duplex and arrow) in each new generation. The MA experiment was conducted for up to 50 consecutive generations.

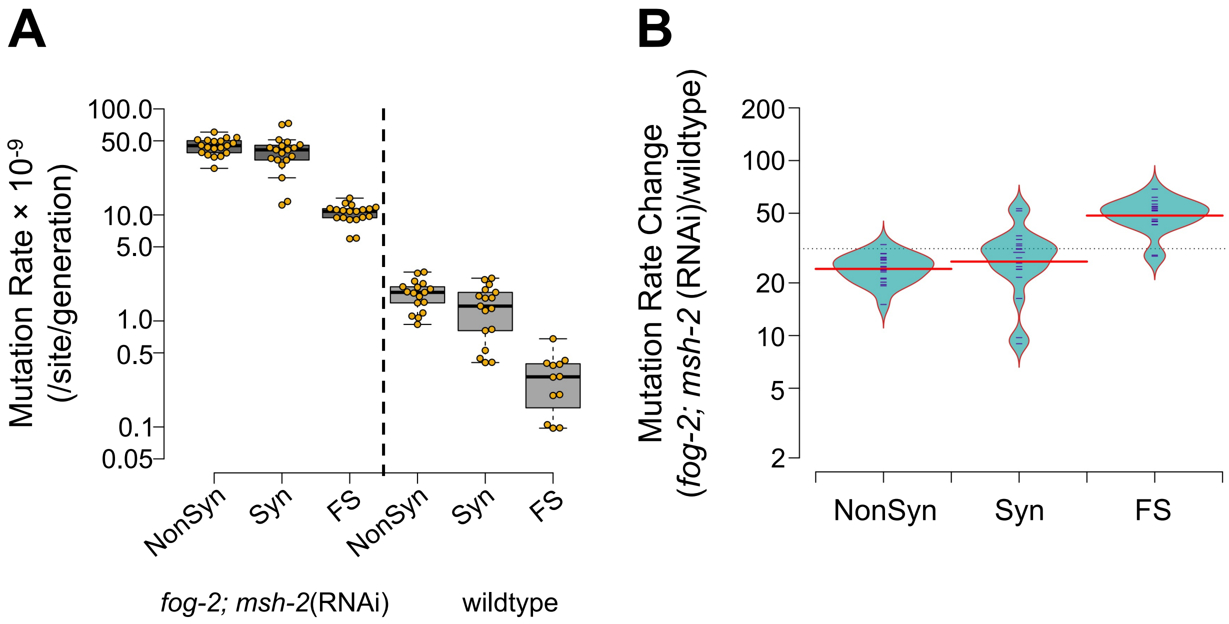

Supplemental Figure S2 Mutation rates in exons of *fog-2(q71)*; *msh-2*(RNAi) knockdown versus wildtype MA lines. For brevity, *fog-2*; *msh-2*(RNAi) refers to *fog-2(q71)*; *msh-2*(RNAi) MA lines. Nonsynonymous, synonymous, and frameshift mutations are referred to as Nonsyn, Syn, and FS in the figures, respectively. (A*)* The synonymous and nonsynonymous substitution rates do not differ significantly in the *fog-2; msh-2*(RNAi) knockdown MA lines. However, frameshift mutations in the *fog-2; msh-2*(RNAi) knockdown MA lines were less frequent than either synonymous or nonsynonymous substitutions. The corresponding mutation rates in wildtype MA lines are shown for comparison (Konrad *et al.* 2019). (B) The frequencies of nonsynonymous, and synonymous substitutions exhibit a similar increase in *fog-2; msh-2*(RNAi) knockdown MA lines relative to wildtype MA. The frameshift mutation rate in *msh-2* MA lines exhibits a greater increase than either nonsynonymous or synonymous rates compared to wildtype MA lines.

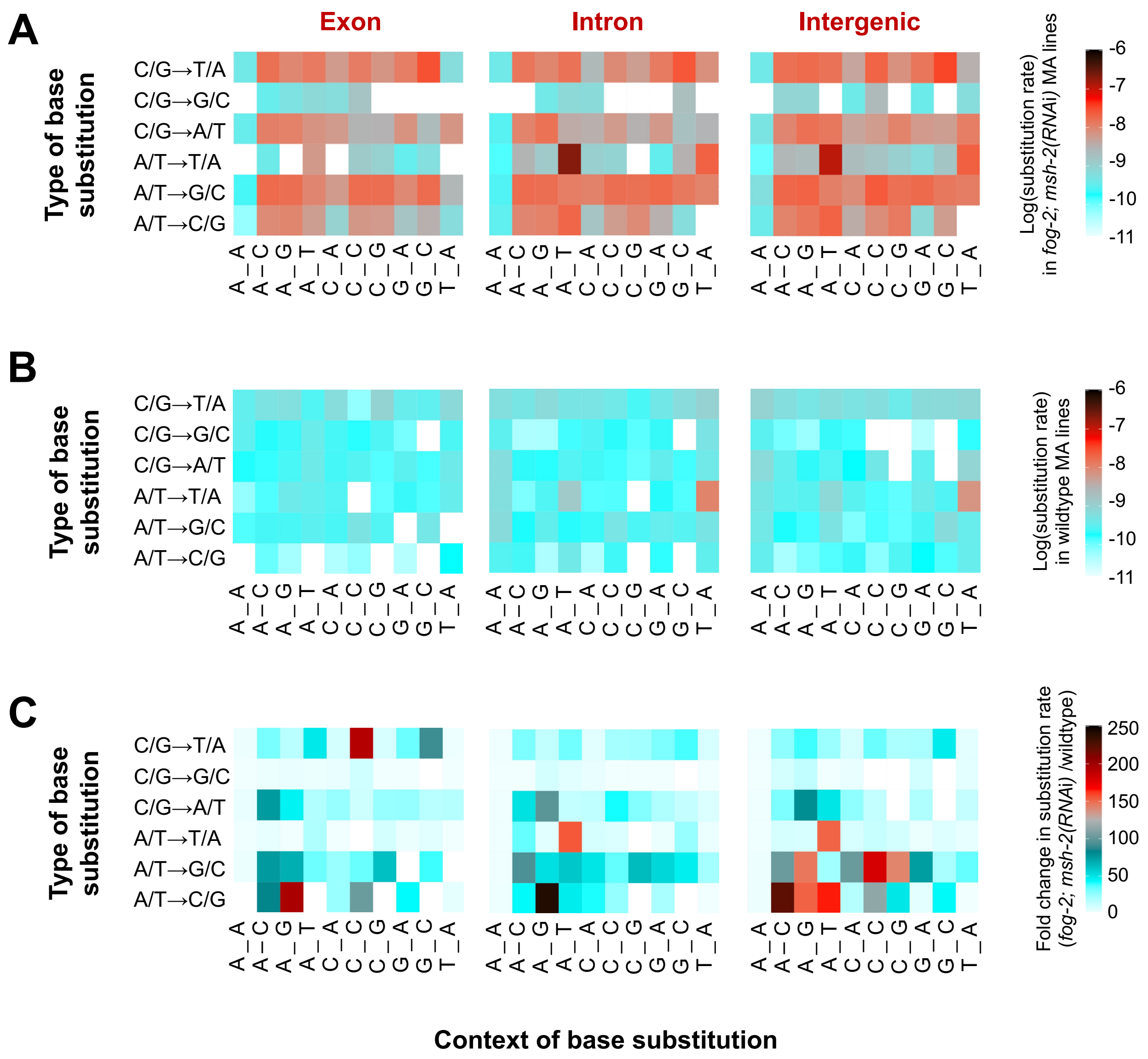

Supplemental Figure S3 Heat maps of specific base substitution rate with their immediate flanking nucleotides. The context-dependent mutational spectra are shown for (A) *fog-2(q71)*; *msh-2*(RNAi) knockdown MA lines and (B) wildtype MA lines. For brevity, *fog-2*; *msh-2*(RNAi) refers to *fog-2(q71)*; *msh-2*(RNAi) knockdown MA lines. While wildtype MA lines exhibit a strong A/T → T/A mutation bias when flanked by a 5′−T and a 3′−A, *fog-2*; *msh-2*(RNAi) knockdown lines exhibit an increase in the same substitution type when flanked by a 5′−A and a 3′−T. (C) The increase in each context-dependent substitution rate in *fog-2*; *msh-2*(RNAi) versus wildtype MA lines differs between exons, introns, and intergenic regions.

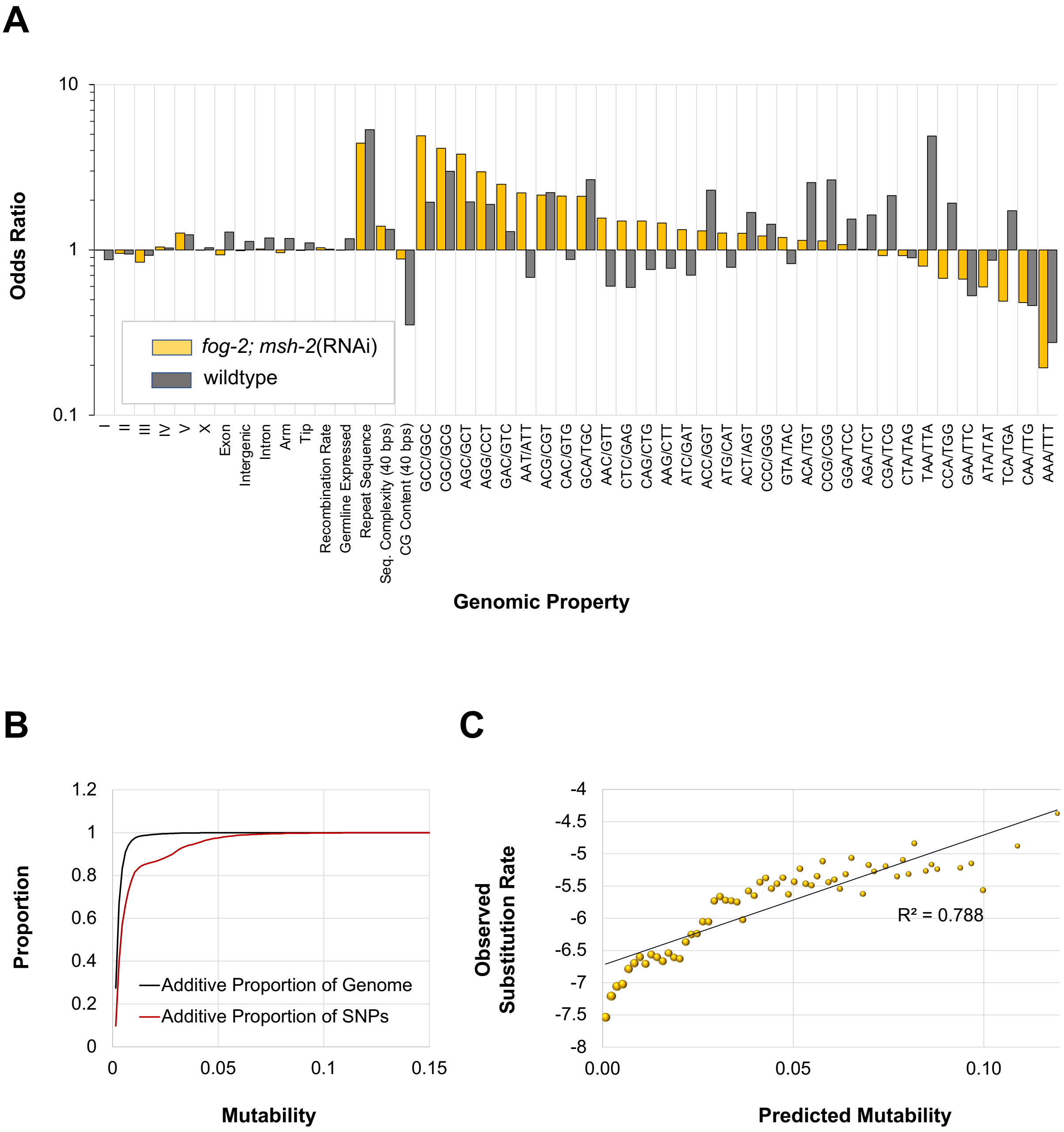

Supplemental Figure S4 Genomic properties associated with base substitution sites. (A) Odds ratios of the contribution of various genomic predictors towards site mutability as estimated by a logistic regression approach. For brevity, *fog-2*; *msh-2*(RNAi) refers to *fog-2(q71)*; *msh-2*(RNAi) MA lines. (B) The cumulative proportion of the genome (black) and SNPs (red) covered by increasing predicted mutabilities. (C) The correlation between predicted mutability and observed mutation rate is significant ($R^2 = 0.79$, $p < 2.2 \times 10^{-16}$).

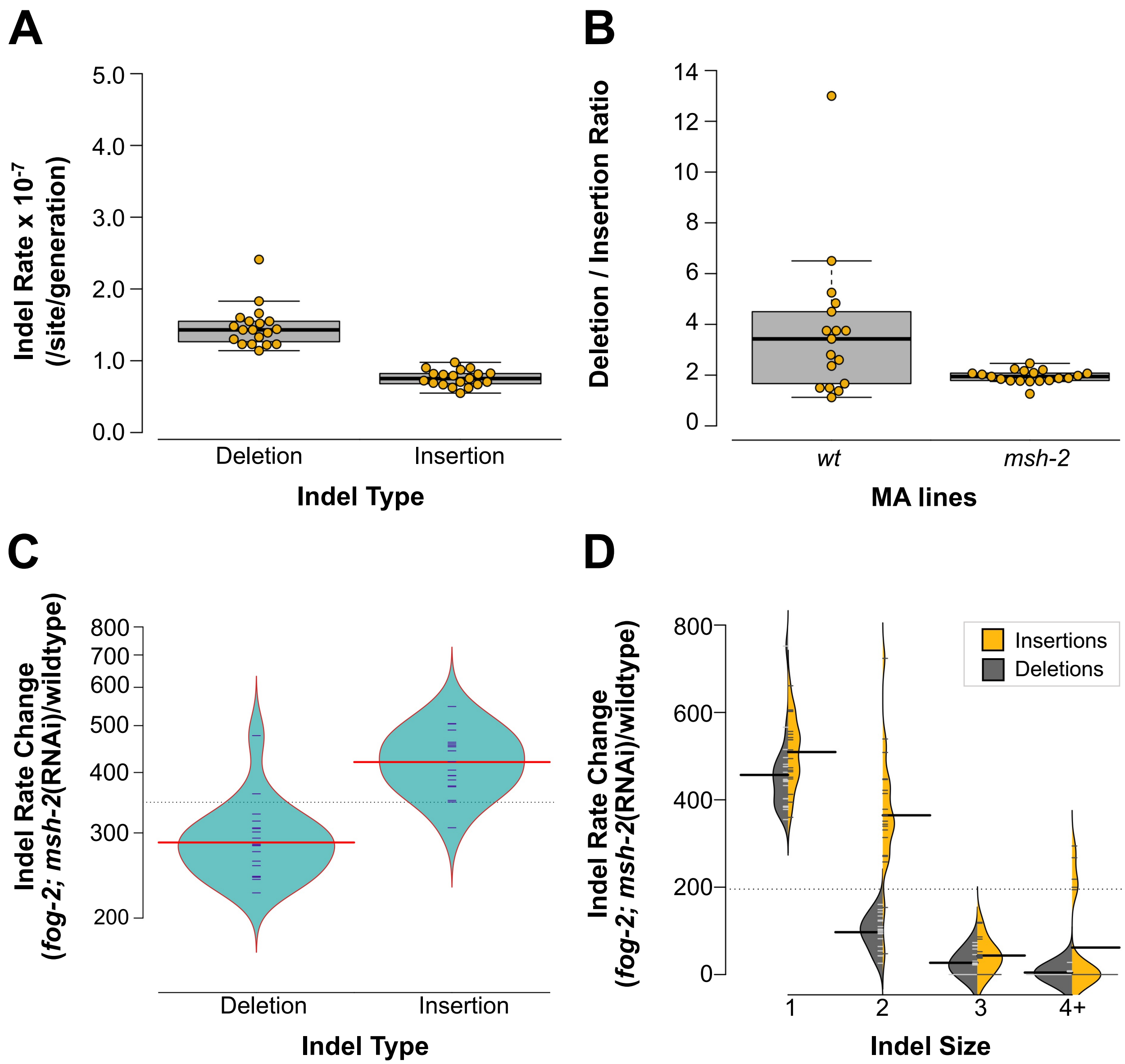

Supplemental Figure S5 Small indel rates in *fog-2(q71)*; *msh-2*(RNAi) knockdown MA lines compared to wildtype MA lines. For brevity, *fog-2*; *msh-2*(RNAi) refers to *fog-2(q71)*; *msh-2*(RNAi) knockdown MA lines in the figures. (A) The deletion rate in the *fog-2*; *msh-2*(RNAi) knockdown MA lines was significantly greater than the insertion rate. (B*) fog-2*; *msh-2*(RNAi) knockdown resulted in a significantly lower ratio of deletions per insertion relative to the wildtype MA lines. (C) The increase in the rate of insertions in *fog-2*; *msh-2*(RNAi) knockdown MA lines relative to wildtype MA lines is significantly greater than the increase in the rate of deletions. (D*)* Both indel type (insertion *vs.* deletion), indel size, and the interaction of indel type and size have significant effects on the increase in indel rate in *fog-2*; *msh-2*(RNAi) knockdown relative to wildtype MA lines. The rate of single base pair indels exhibits the greatest increase in *fog-2*; *msh-2*(RNAi) knockdown MA lines, followed by 2 bp indels. Insertions increase more than deletions, especially the 2 bp insertions.

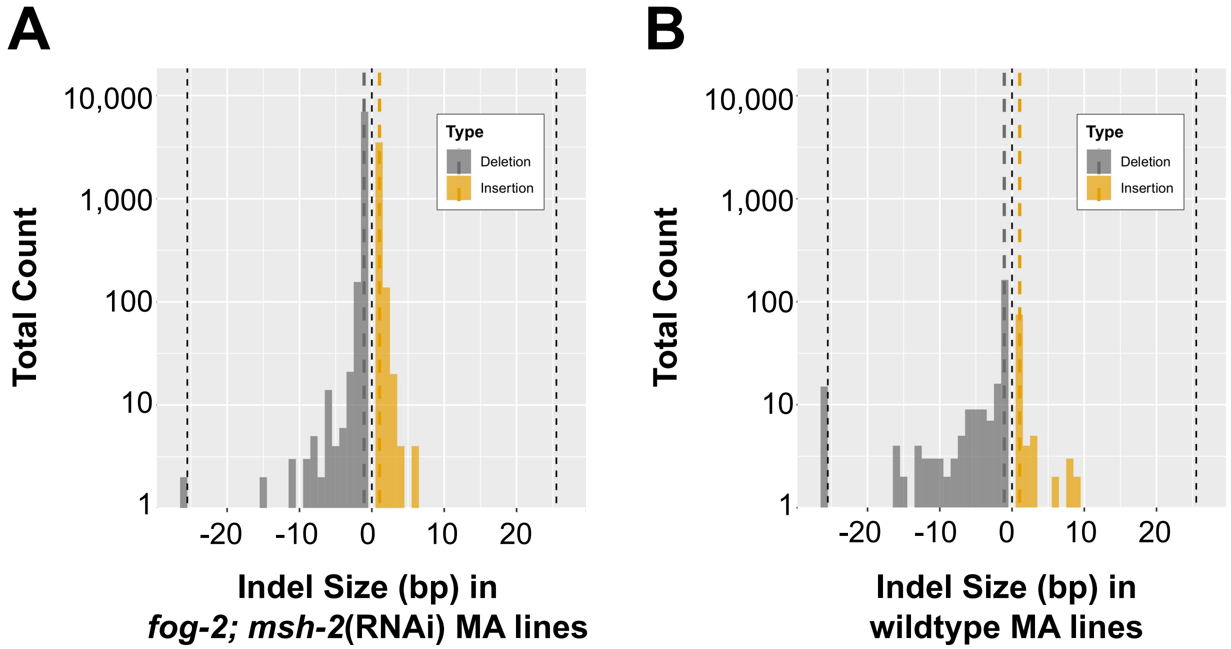

Supplemental Figure S6 The size distribution of small indels. (A) The size distributions of both deletions and insertions in *fog-2(q71)*; *msh-2*(RNAi) knockdown MA lines display a preponderance of single nucleotide indels. There was no difference in the average size of insertions (1.06 bp) and deletions (1.08 bp). For brevity, *fog-2*; *msh-2*(RNAi) in the figure refers to *fog-2(q71)*; *msh-2*(RNAi) knockdown MA lines. (B) In contrast, the average deletion size (6.38 bp) was significantly greater than the average insertion size (1.94 bp) in the wildtype MA lines.

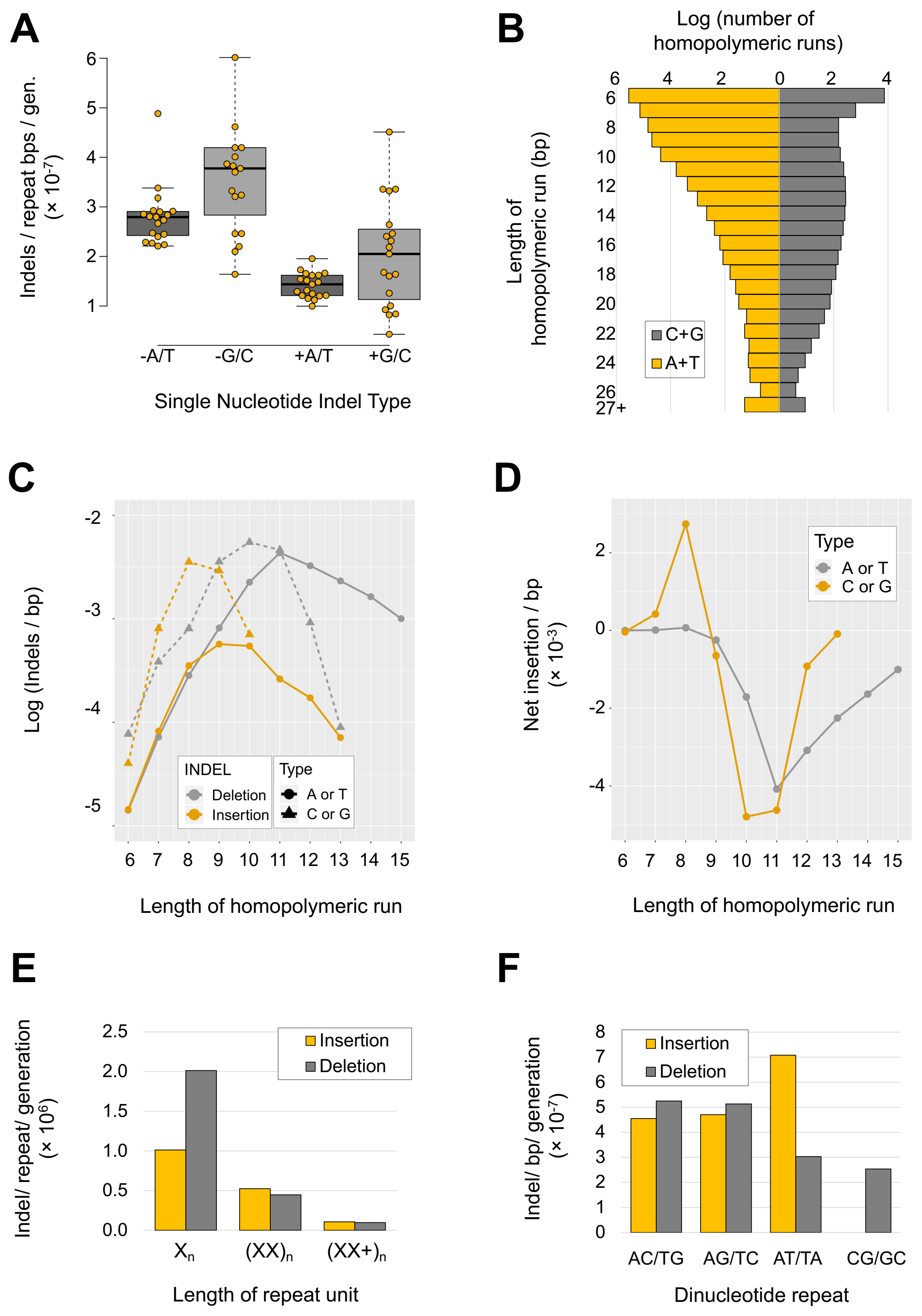

Supplemental Figure S7 Variation in indel frequencies within indel repeats in the *fog-2(q71)*; *msh-2*(RNAi) knockdown MA lines. (A) G/C homopolymeric runs of ≥6 bps in length have a

higher indel rate per base per generation than A/T runs. Both indel type (deletions *vs.* insertions) and nucleotide (A+T versus C+G), have significant effects on the per base indel rate in homopolymeric runs. (B) The frequency and length distribution of homopolymeric runs depends on the type of nucleotide comprising the homopolymer. *(C)* The indel rates in homopolymeric runs vary with the length and type of nucleotide in the runs. Both insertion and deletion rates increase with the length of a run up to a point, and subsequently decline. The deletion rates were highest in runs of 10-11 bps for both A/T and G/C and lower for runs that were either smaller or larger. The insertion rates were highest when the length of a run was 8-9 bps for G/C and 8-10 bps for A/T. (D*)* The net gain and loss rates in homopolymeric runs vary with length and composition. The greatest net loss was in runs of 10-11 bps for both A/T and G/C. The greatest net gain was for G/C runs of 8 bps. (E) Deletions per repeat are only significantly higher than insertions in homopolymeric runs. In di- and polynucleotide repeats, insertions are roughly equal in frequency. (F) While most di-nucleotide repeats had a slightly higher deletion rate than insertion rate, AT dinucleotide repeats had an insertion rate that was almost twice as high as their deletion rate.

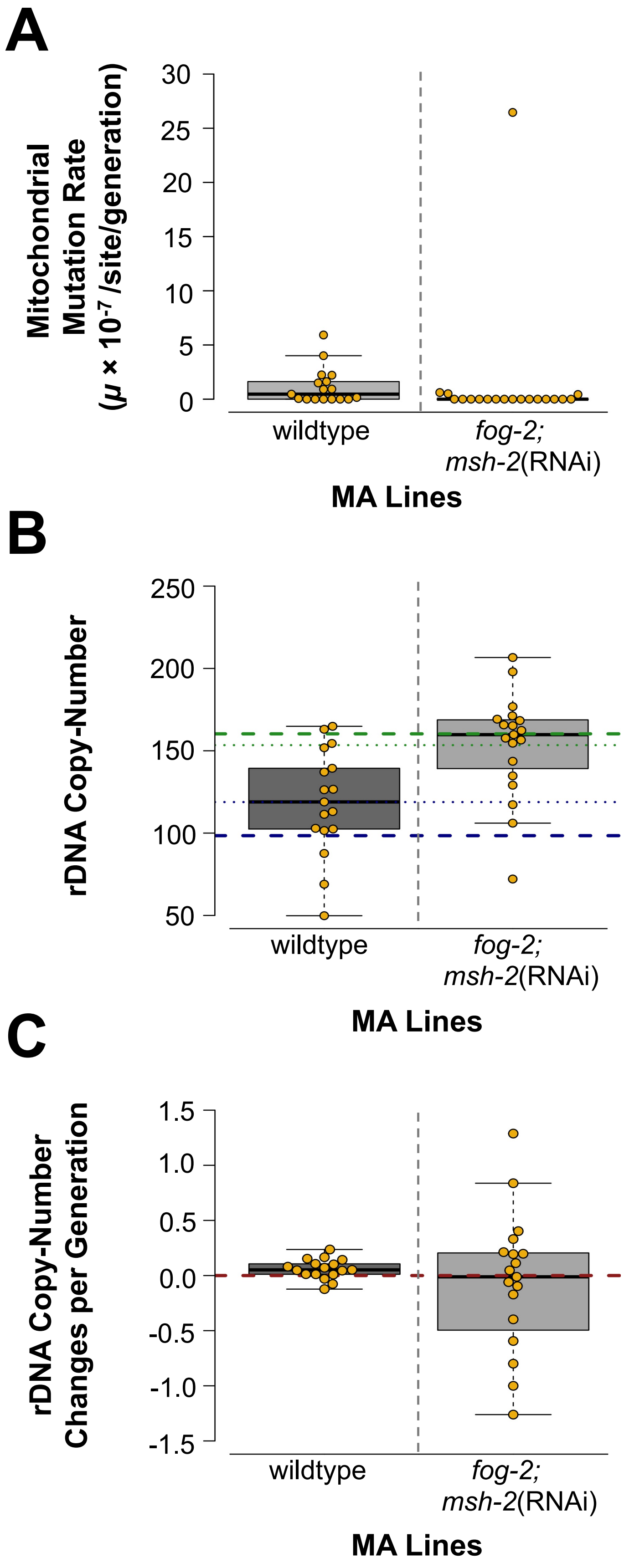

Supplemental Figure S8 Comparisons between the *fog-2(q71)*; *msh-2*(RNAi) knockdown and wildtype MA lines with respect to mitochondrial mutations, and changes in rDNA copy-number. For brevity, *fog-2*; *msh-2*(RNAi) in the figure refers to *fog-2(q71)*; *msh-2*(RNAi) knockdown MA lines. (A) Line-specific mtDNA mutation rate in the *fog-2*; *msh-2*(RNAi) knockdown versus wildtype MA experiments. Due to the large number of *fog-2*; *msh-2*(RNAi) knockdown MA lines with no mitochondrial mutations and a substantial difference in the number of generations, a statistical test of the difference in mtDNA mutation rates between wildtype and *fog-2*; *msh-2*(RNAi) knockdown MA lines was not performed. (B) rDNA copy-number increased by an average 20 copies in the wildtype MA lines whereas *fog-2*; *msh-2*(RNAi) knockdown MA lines exhibited no significant change in rDNA copy-number relative to their ancestral state. The blue and green dashed line displays the estimated rDNA copy-number in the N2 wildtype and *fog-2(q71lf)* ancestral strain, respectively. (C) The variation in the rDNA copy-number change per generation relative to the ancestral state is significantly greater in the *fog-2*; *msh-2*(RNAi) knockdown than in the wildtype MA lines.

LITERATURE CITED

Belfield, E. J., Z. J. Ding, F. J. C. Jamieson, A. M. Visscher, S. J. Zheng *et al.,* 2018 DNA mismatch repair preferentially protects genes from mutation. Genome Res. 28: 66–74. https://doi.org/10.1101/gr.219303.116

Dillon, M. M., W. Sung, R. Sebra, M. Lynch, and V. S. Cooper, 2017 Genome-wide biases in the rate and molecular spectrum of spontaneous mutations in *Vibrio cholerae* and *Vibrio fischeri*. Mol. Biol. Evol. 34: 93–109. https://doi.org/10.1093/molbev/msw224

Konrad, A., M. J. Brady, U. Bergthorsson, and V. Katju, 2019 Mutational landscape of spontaneous base substitutions and small indels in experimental *Caenorhabditis elegans* populations of differing size. Genetics 212: 837–854. https://doi.org/10.1534/genetics.119.302054

Lang, G. I., L. Parsons, and A. E. Gammie, 2013 Mutation rates, spectra, and genome-wide distribution of spontaneous mutations in mismatch repair deficient yeast. G3 3: 1453–1465. https://doi.org/10.1534/g3.113.006429

Lee, H., E. Popodi, H. Tanga, and P. L. Foster, 2012 Rate and molecular spectrum of spontaneous mutations in the bacterium *Escherichia coli* as determined by whole-genome sequencing. Proc. Natl. Acad. Sci. USA 109: E2774–2783. https://doi.org/10.1073/pnas.1210309109

Long, H., S. F. Miller, C. Strauss, C. Zhao, L. Cheng *et al.*, 2016 Antibiotic treatment enhances the genome-wide mutation rate of target cells. Proc. Natl. Acad. Sci. USA 113: E2498–E2505. https://doi.org/10.1073/pnas.1601208113

Long, H., S. F. Miller, E. Williams, and M. Lynch, 2018 Specificity of the DNA mismatch repair system (MMR) and mutagenesis bias in bacteria. Mol. Biol. Evol. 35: 2414–2421. https://doi.org/10.1093/molbev/msy134

Lujan, S. A., A. R. Clausen, A. B. Clark, H. K. MacAlpine, D. M. MacAlpine *et al.* 2014 Heterogeneous polymerase fidelity and mismatch repair bias genome variation and composition. Genome Res. 24: 1751–1764. https://doi.org/10.1101/gr.178335.114

Ossowski, S., K. Schneeberger, J. L. Lucas-Lledó, N. Warthmann, R. M. Clark *et al.* 2010 The rate and molecular spectrum of spontaneous mutations in *Arabidopsis thaliana*. Science 327: 92–94. https://doi.org/10.1126/science.1180677

Serero, A., C. Jubin, S. Loeillet, P. Legoix-Né, and A. G. Nicolas, 2014 Mutational landscape of yeast mutator strains. Proc. Natl. Acad. Sci. USA 111: 1897–1902. https://doi.org/10.1073/pnas.1314423111

Sung, W., M. S. Ackerman, J. F. Gout, S. F. Miller, E. Williams *et al.*, 2015 Asymmetric context-dependent mutation patterns revealed through mutation-accumulation experiments. Mol. Biol. Evol. 32: 1672–1683. https://doi.org/10.1093/molbev/msv055

Zhu, Y. O., M. L. Siegal, D. W. Hall, and D. A. Petrov, 2014 Precise estimates of mutation rate and spectrum in yeast. Proc. Natl. Acad. Sci. USA 111: E2310– E2318. https://doi.org/10.1073/pnas.1323011111
